# The Immunomodulatory Effects of Active Ingredients From *Nigella sativa* in RAW264.7 Cells Through NF-κB/MAPK Signaling Pathways

**DOI:** 10.3389/fnut.2022.899797

**Published:** 2022-05-31

**Authors:** Jinfeng Wei, Baoguang Wang, Yixiao Chen, Qiuyi Wang, Adel F. Ahmed, Yan Zhang, Wenyi Kang

**Affiliations:** ^1^National R & D Center for Edible Fungus Processing Technology, Henan University, Kaifeng, China; ^2^Shenzhen Research Institute of Henan University, Shenzhen, China; ^3^Joint International Research Laboratory of Food & Medicine Resource Function, Henan Province, Kaifeng, China; ^4^Medicinal and Aromatic Plants Researches Department, Horticulture Research Institute, Agricultural Research Center, Giza, Egypt; ^5^Hebei Food Safety Key Laboratory, Hebei Food Inspection and Research Institute, Shijiazhuang, China

**Keywords:** *Nigella sativa*, immunoregulation, NF-κB, MAPK, RAW264.7 cells

## Abstract

*Nigella sativa* is a valuable herb for its functional compositions in both food and medication. *N. sativa* seeds can enhance immunity, anti-inflammation and analgesia and hypoglycemia, but most of the related researches are related to volatile oil and extracts, and the activity and mechanism of compounds is not clear. In this study, Ethyl-α-D-galactopyranoside (EG), Methyl-α-D-glucoside (MG), 3-*O*-[β-*D*-xylopyranose-(1 → 3)-α-*L*-rhamnose-(1 → 2)-α-*L*-arabinose]-28-*O*-[α-*L*-rhamnose-(1 → 4)-β-*D*-glucopyranose-*L*-(1 → 6)-β-*D*-glucopyranose]-hederagenin (HXRARG) and 3-*O-*[β-*D*-xylopyranose-(1 → 3)-α-*L*-rhamnose-(1 → 2)-α-*L*-arabinose]-hederagenin (HXRA) were isolated and identified from *N. sativa* seeds. In addition, four compounds could activate NF-κB pathway by promoting the expression of phosphorylation of P65 and IκBα, promoting the phosphorylation of JNK, Erk and P38 to activate MAPK signaling pathway, enhancing the proliferation and phagocytic activity of RAW264.7 cells, and promoting the release of NO, TNF-α and IL-6 on RAW264.7 cell *in vitro*. The results showed that *N. sativa* can be used as dietary supplement to enhance immune.

## Introduction

Hippocrates put forward the creed of “make food your medicine, and make medicine be your food” nearly 2, 500 years ago (1, 2). This creed revived interest and spawned terms such as pharmaceuticals, nutritional medicines and functional foods. These terms are not only becoming more and more popular among researchers, but also arouse consumer interest in health regimens based on a natural diet. In recent decades, our understanding of the basic principles and correlation between nutrition and human health has been greatly enhanced (3, 4). It is increasingly clear that diet-based natural compounds represent the basic categories of regulatory factors that affect human health (5). Historical evidence in the Chinese traditional medical system emphasizes the role of natural dietary factors in health and disease management, and natural products can be used as immune enhancers to improve the quality of life (6, 7). In addition, the safety, effectiveness and affordability of nutritional drugs stimulate consumer confidence. As a consequence, the global focus is on the study of bioactive natural regulators for human health. This seems to be more important in modern times, because a diet-dependent preventive lifestyle may reduce our growing dependence on antibiotics or may combine to increase the efficacy of antibiotics (8).

Immune system is an important part of the body. It is a defense system for the body to resist pathogen infection, avoid the invasion of various external dangerous invaders, maintain the stability of internal environment, and monitor the mutation of its own cells (9). Besides, immunity can be subdivided into native immunity and adaptive immunity according to the way of acquisition. Native immunity is an innate ability of the human body to resist foreign infections in a non-specific manner. (10). Moreover, adaptive immunity is the body's third line of defense to protect itself, characterized by its protection function only against a specific pathogen. Macrophages are key innate immune cells found in practically all of the body's major organs (11). They are the initial line of defense against infections and cancerous cells infiltrating the body (12, 13). Macrophages drive other immune cells to the infected location, where they produce cytokines, chemokines, and inflammatory chemicals to stimulate inflammation (14, 15). In addition, macrophages initiate adaptive immune responses through antigen presentation and cytokine secretion. As a consequence, macrophages are considered to be some key target cells for immune regulation (16).

Pattern recognition receptors (PRR) is a protein that interacts with the innate immune system and is responsible for identifying pathogen and antigen molecular patterns (17). When PRR are activated, intracellular signaling cascades like as mitogen-activated protein kinases (MAPKs) and nuclear factor (NF)-κB are subsequent activated, resulting in gene transcription that is involved in the inflammatory response (18, 19). Macrophages are activated in this process, which results in greater phagocytosis, enhanced synthesis of immunomodulatory signal molecules like nitric oxide (NO), interleukin-6 (IL-6), tumor necrosis factor-α (TNF-α) and reactive oxygen species (ROS) (20). RAW264.7 cells are utilized to investigate how different medications or immunomodulators affect immune response, mostly to detect the proliferation ability, phagocytosis ability, secretion level of various cytokines and killing effect on tumor cells of RAW264.7 cells, and to evaluate the regulatory response of some immunomodulators to initial immunity (21, 22).

*Nigella sativa* belongs to Ranunculaceae family that can be found in southern Europe, southwest Asia, and North Africa (23). Anti-cancer, bacteriostasis, anti-hypertension, liver protection, anti-inflammation, antipyretic, analgesia, and elimination of ROS are just a few of the biological properties of *N. sativa* seeds. So, they are widely used in food and medicine (24, 25). Alshatwi A.A found that *N. sativa* seeds have immunostimulatory effect on the growth of human peripheral blood monon-uclear cells triggered by non-phytohemagglutinin (26). Ebaid H found *N. sativa* oil could slightly improve the damaged spleen tissue, repair thymus tissue in a time-dependent manner, and restore cellular and humoral immunity (27).

**Figure 1:**
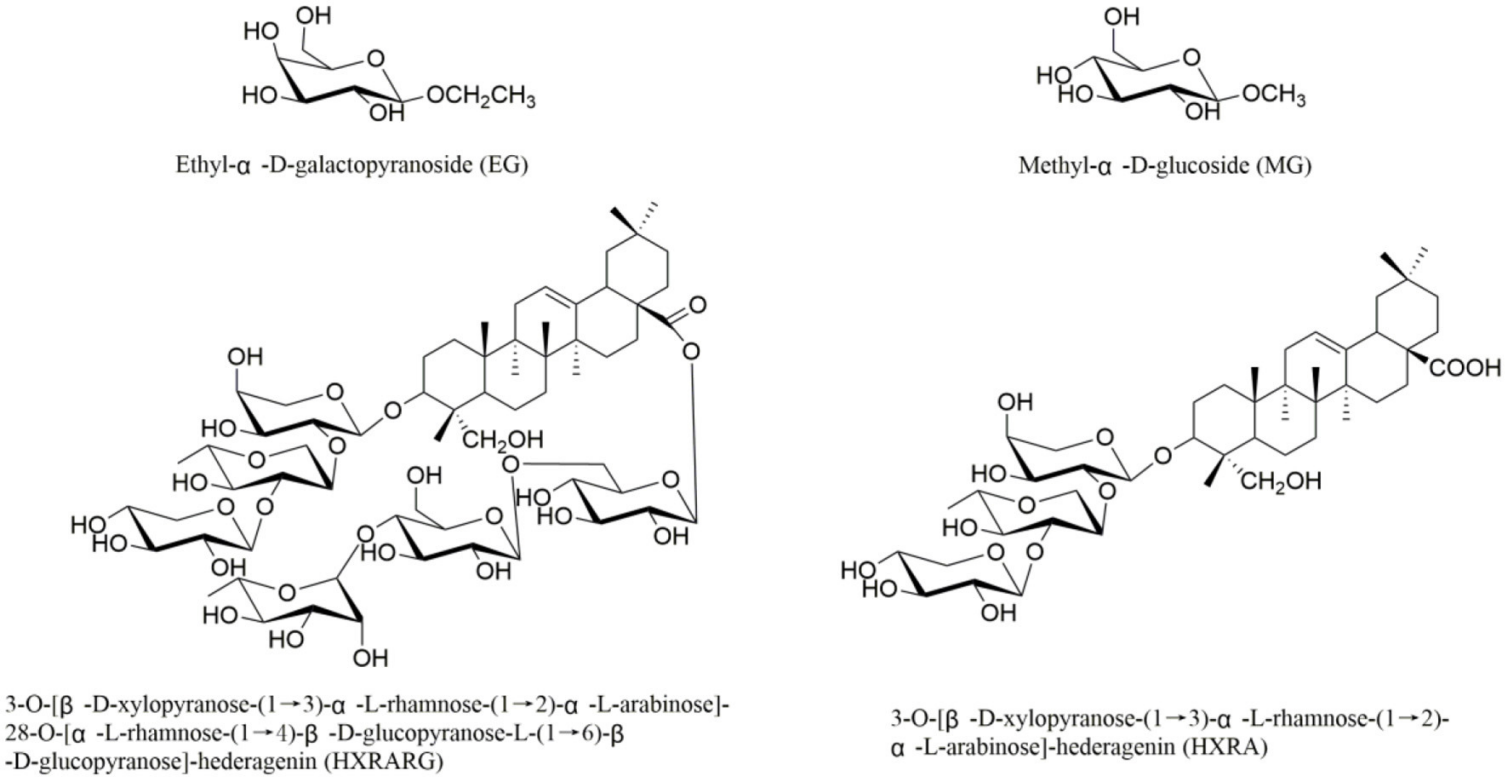
Structures of compounds EG, MG, HXRARG, and HXRA from *Nigella sativa*.

Our ongoing research (28–31), four compounds, (Figure 1) Ethyl-α-D-galactopyranoside (EG), Methyl-α-D-glucoside (MG), 3*-O*-[β-*D*-xylopyranose-(1 → 3)-α-L-rhamnose-(1 → 2)-α-*L*-arabinose]-28-*O*-[α-*L*-rhamnose-(1 → 4)-β-*D*-glucopyranose-L-(1 → 6)-β-*D*-glucopyranose]-hederagenin (HXRARG) and 3-*O*-[β-*D*-xylopyranose-(1 → 3)-α-L-rhamnose-(1 → 2)-α-*L*-arabinose]-hederagenin (HXRA), were isolated and identified and they were found to stimulate NO production in RAW264.7 cells. It is speculated that EG, MG, HXRA and HXRARG have immunomodulatory effects. According to the literatures, although pharmacological studies have shown that *N. sativa* seeds have immune enhancement, but there is no study on the active compounds. Thus, in this paper, the immune activity and mechanism of EG, MG, HXRARG and HXRA were carried out.

## Materials and Methods

### Chemicals and Reagents

The following reagents were used in this research: Mouse macrophage cell line RAW264.7 was purchased from the Cell Bank of the Chinese Academy of Sciences (Shanghai, China); Dulbecco's Modified Eagle's Medium (DMEM) and neutral red staining solution were bought from Solarbio (Beijing, China); Lipopolysaccharide (LPS) were obtained from Sigma-Aldrich (St. Louis, MO, USA); Griess kit and ROS kit were purchased from Beyotime Biotechnology (Shanghai, China); Mouse TNF-α and IL-6 ELISA kit were purchased from Beijing 4A Biotech Co, Ltd (Beijing, China); Primer iNOS, TNF-α, IL-6 and Cox-2 were designed and synthesized by Thermo Fisher Scientific (Shanghai, China Thermo Fisher scientific (Shanghai, China); *Evo M-MLV* RT Kit with gDNA Clean and TB Green TM Ex TaqTM II (Tli RNadeH Plus), Bulk kit were obtained from TaKaRa; anti-NF-κB p65 antibody, anti-phospho-NF-κB p65 antibody, anti-iNOS antibody, anti-COX-2 antibody, anti-IκBα antibody, anti-phospho-IκBα antibody, anti-P38 MAPK antibody, anti-phospho-P38 MAPK antibody, anti-Erk antibody, anti-phospho-Erk antibody, anti-JNK antibody and anti-phospho-JNK antibody were bought from Cell Signaling Technology (Danvers, MA, USA).

### Cell Culture

RAW264.7 cells were cultured by the assay of Senye Wang (32). At 37°C in a humidified incubator with a 5% CO2 atmosphere, cells were grown in DEME high glucose medium with 10% fetal bovine serum (FBS, Gibco, USA), 100 U/mL penicillin and 100 μg/mL streptomycin. LPS was adopted as a positive control.

### Cell Viability Assay

Cell viability assay was consistent with the method previously reported (33). Briefly, In 96-well-plates, 1.0 × 10^4^ cells were plated. EG, MG, HXRARG and HXRA were added at increasing concentrations over the next 24 h. Each well-received 20 μL of MTT reagent 4 h before the end of the experiments. To dissolve the formazan, 150 μL of dimethyl sulfoxide (DMSO) was added at the end. At a wavelength of 570 nm, absorbance was recorded.

### Phagocytic Activity

The phagocytic capacity of macrophages was measured using the previously described approach (34). RAW264.7 cells were seeded at a density of 1.0 × 10^4^ cells per well in 96-well-plates and incubated overnight. RAW264.7 cells were incubated with increasing concentrations of EG (80, 60 and 40 μM), MG (80, 60 and 40 μM), HXRARG (80, 60 and 40 μM), HXRA (8, 6 and 4 μM) or LPS (1 μg/mL) for 24 h. We discarded the old culture medium, washed gently with PBS solution for 2–3 times, 100 μL of 0.1 percent neutral red solution was added to each well, and cultivation was continued for 30 min. After being washed with PBS solution for three times, the cells were photographed under fluorescence microscope. Each well-added cell lytic solution comprising acetic acid and anhydrous ethanol (1:1, V/V) and was kept at 4°C overnight. A micro-plate reader was used to measure the absorbance of each hole at 550 nm.

### Measurement of NO

RAW264.7 cells were seeded at a density of 1.0 × 10^5^ cells per well in 24-well-plates overnight. Then RAW264.7 cells were treated with the above methods for 24 h. After culture, the medium (50 μL) was transferred to a new 96-well-plate, 50 μL Griess reagent I and II were added, and the absorbance of each well was measured at 540 nm.

### Determination of IL-6 and TNF-α

For 24 h, RAW264.7 cells were pre-incubated at a density of 1.5 × 10^5^ cells per well in 24-well-plates, and then cultured with different concentrations of EG (80, 60, and 40 μM), MG (80, 60, and 40 μM), HXRARG (80, 60, and 40 μM), HXRA (8, 6, and 4 μM) or LPS (1 μg/mL) for 24 h. The cell culture medium was centrifuged using ELISA kit to determine the concentration of TNF-α and IL-6.

### Determination of Intracellular ROS

RAW264.7 cells were seeded at a density of 5.0 × 10^4^ cells per well on a 6-well plate overnight, and then each well was exchanged with DMEM medium containing different concentrations of EG (80, 60, and 40 μM), MG (80, 60, and 40 μM), HXRARG (80, 60 and 40 μM), HXRA (8, 6, and 4 μM) or LPS (1 μg/mL). After gently washing the cells with serum-free DMEM, the 20 min was incubated with 10 μM DCFH-DA and flipped every 3–5 min, so that the DCFH-DA could be in full contact with the cells. Subsequently, after passing through a 300 mesh nylon screen, flow cytometry was used to identify the change in ROS.

### Western Blot

The cells after treatment for 24 h using the above experimental procedure, and Western Blot experiment was conducted according to the reference (31). Total protein was extracted using the proper volume of a weak radioimmune precipitation assay (RIPA) lysis buffer based on the density of the cultivated cells (about 100 μL per well). The BCA method was utilized to determine protein concentrations 0.20 μg of proteins from various groups were separated by 12% SDS-polyacrylamidegel electrophoresis (PAGE) and transferred onto polyvinylidene difluoride (PVDF) membrane after boiling at 100 °C for 5 min. The PVDF membranes were exposed with the relevant primary antibodies at 4°C overnight after being blocked with 5 percent dry skimmed milk, and then treated with the corresponding HRP-linked secondary antibodies at room temperature for 2 h. Enhanced chemiluminescence (ECL) detection reagent was performed to visualize the protein bands, which were scanned using ImageJ software.

### Quantitative Real-time PCR (qRT-PCR)

In the same way as the previous cell processing approaches, then qRT-PCR were used as previously reported (35). In brief, RAW264.7 cells were inoculated for 12 h on 6-well-plates and then treated for 24 h with LPS or different doses of EG, MG, HXRARG, and HXRA. Total RNA was extracted using Trizol reagent and reversed to cDNA using the *Evo M-ML V* RT Kit with gDNA Clean, following the manufacturer's instructions. A spectrophotometer was used to calculate the concentration of extracted RNA. The SYBR Green *Pro Taq* HS Premix qPCR kit was used to detect the expression of mRNAs. β-actin was assessed as on normalization control and the sequences were listed in Table 1.

**Table 1:** Primers sequence.

**Name**	**Primer**	**Sequence (5^**′**^ → 3^**′**^)**
COX-2	Forward	GGGCTCAGCCAGGCAGCAAAT
	Reverse	GCACTGTGTTTGGGGTGGGCT
iNOS	Forward	GCTCGCTTTGCCACGGACGA
	Reverse	AAGGCAGCGGGCACATGCAA
IL-6	Forward	AGACAAAGCCAGAGTCCTTCAGAGA
	Reverse	GCCACTCCTTCTGTGACTCCAGC
TNF-α	Forward	CCCTCCTGGCCAACGGCATG
	Reverse	TCGGGGCAGCCTTGTCCCTT
β-actin	Forward	AGCCTTGTAGGTACCCAACC
	Reverse	TCCACTCACCTGAGGTGCTGAA

### Statistical Analysis

The numerical statistics were examined using one-way ANOVA in SPSS 19.0 software, and the experimental results were reported as mean standard deviation. The software GraPhPad Prism 5.0 was used to analyze all of the column images.

## Results

### Effects of EG, MG, HXRARG, and HXRA on Cell Viability of RAW264.7 Cells

**Figure 2:**
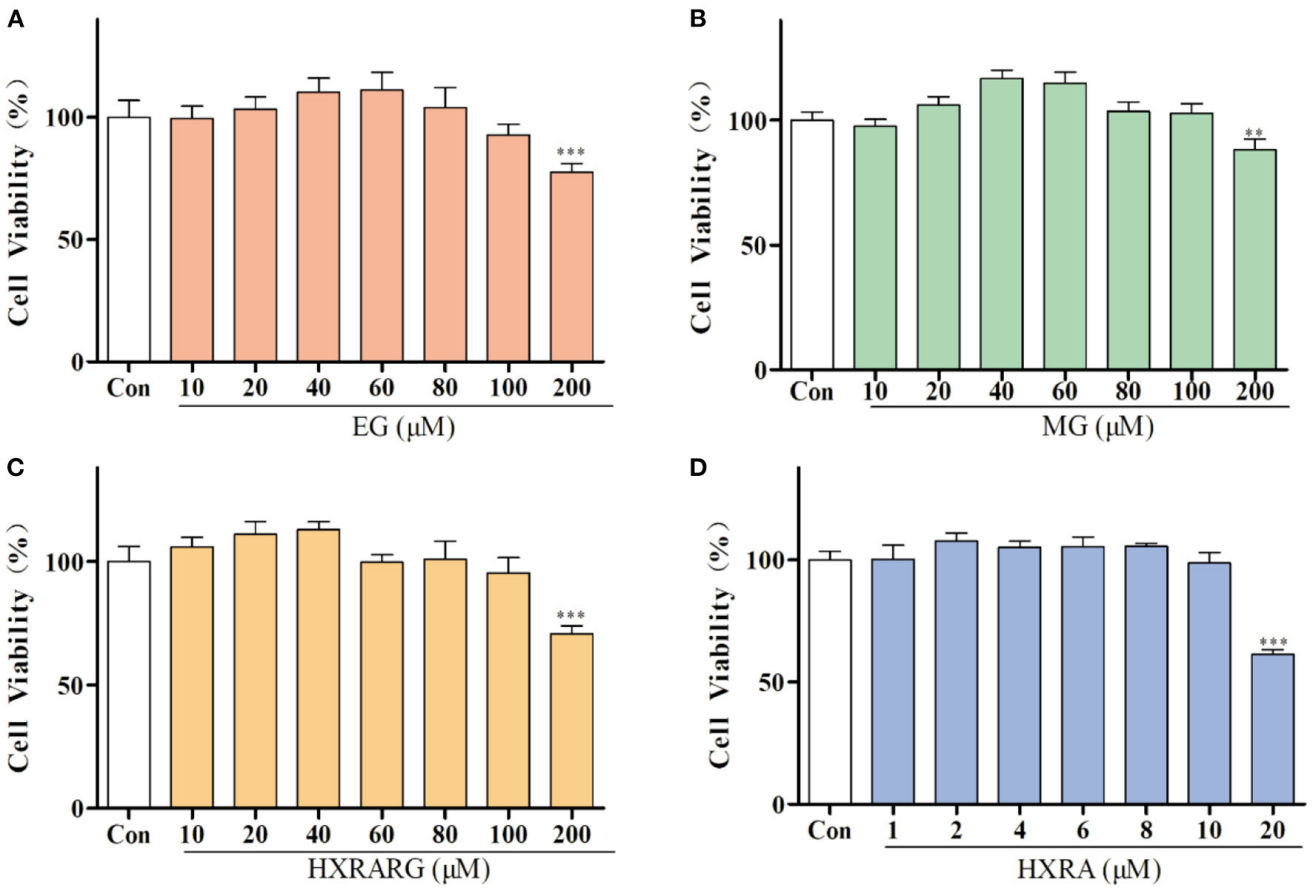
Effects of EG **(A)**, MG **(B)**, HXRARG **(C)**, and HXRA **(D)** on the viability of RAW264.7 cells. Data are expressed as the means ± SD. Compared with the control, ^**^P < 0.01, ^***^P < 0.001.

Compared with the control group, EG, MG or HXRARG with 200 μM could significantly inhibit the proliferation of macrophages, while the concentration of 40–80 μM could promote the proliferation of RAW264.7 cells (Figures 2A–C). After treated with HXRA (20 μM) for 24 h, the survival rate of RAW264.7 macrophages was only about 50%, but when the concentration was 2–10 μM, it could promote the proliferation of RAW264.7 cells (Figure 2D). Macrophages that have been activated serve a vital part in the immune response, and their proliferative activity can be adopted as an index of immunoregulation and cytotoxicity. So, 80, 60, and 40 μM were selected as the drug concentrations of EG, MG, HXRARG and 8, 6, and 4 μM were selected as the concentrations of HXRA in the next experiments.

### Effects of EG, MG, HXRARG, and HXRA on Phagocytic Function of RAW264.7 Cells

**Figure 3:**
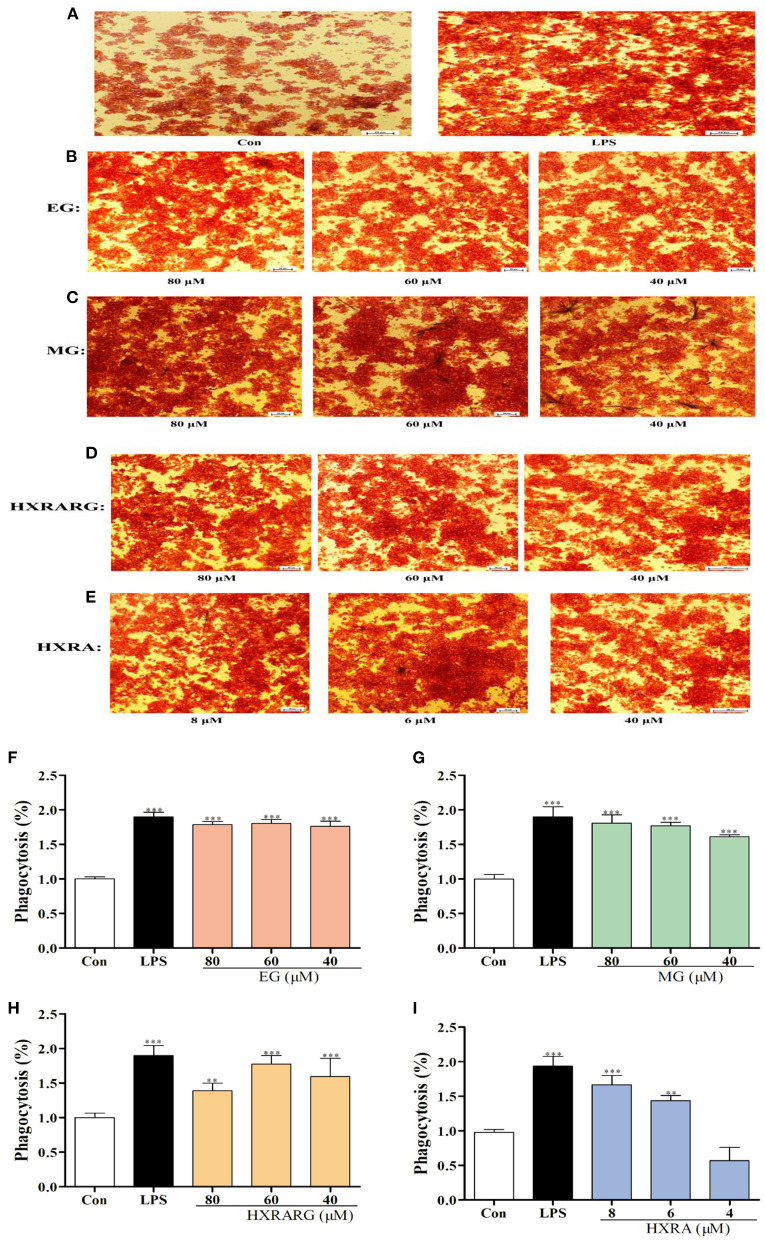
Effects of EG, MG, HXRARG, and HXRA on phagocytosis of neutral red in RAW264.7 cells. **(A–E)** microscope picture, **(F–I)** quantitative analysis picture. Data are expressed as the means ± SD. Compared with the control, ^**^P < 0.01, ^***^P < 0.001.

Compared with the control group, after being stimulated by EG, MG, HXRARG, or HXRA, more neutral red enters the cells (Figures 3A–E), indicating that EG, MG, HXRARG, HXRA, or LPS could enhance the phagocytic ability of macrophages. In addition, under the microscope, the volume of RAW264.7 cells increased after LPS treatment, showing irregular polygons and some cells protruding dendritic pseudopodia. The morphological changes of EG, MG, HXRARG or HXRA group were similar to those of LPS group, but the proportion of amoeba cells was significantly lower than that of LPS group. The morphological changes of macrophages may mean the enhancement of cell adhesion to matrix and phagocytic activity to pathogenic microorganisms. The phagocytic index of the LPS group was nearly double that of the control group after cell lysis, as expected. Compared with the control group, except HXRA (4 μM), the ability of RAW264.7 cells to phagocytize when exposed to various doses EG, MG, HXRARG and HXRA was significantly enhanced (Figures 3F–I), which was consistent with the results of microscope photography.

### Effects of EG, MG, HXRARG, and HXRA on No Secretion of RAW264.7 Cells

**Figure 4:**
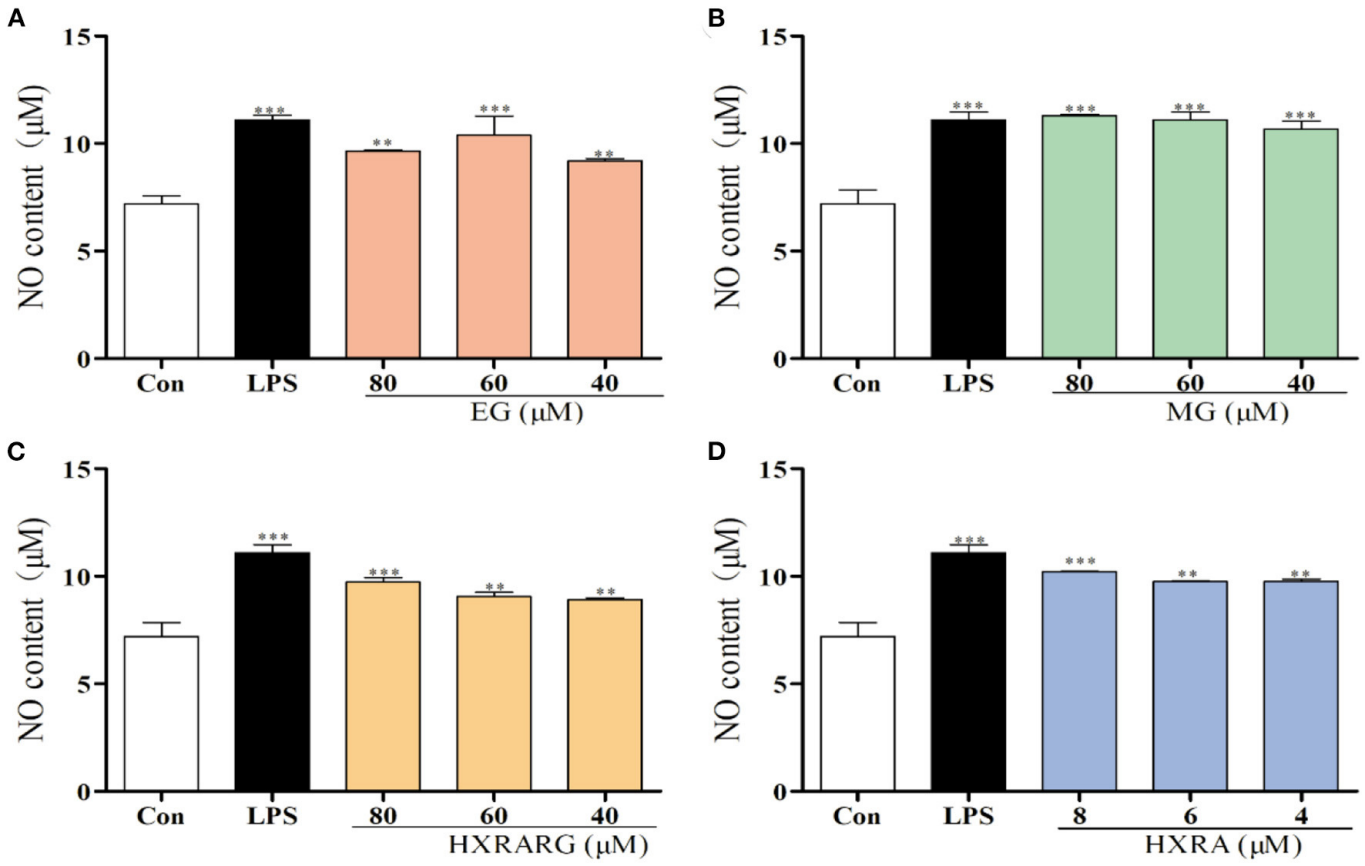
Effects of EG **(A)**, MG **(B)**, HXRARG **(C)**, and HXRA **(D)** on nitric oxide (NO) production in RAW264.7 cells. Data are expressed as the means ± SD. Compared with the control, ^**^P < 0.01, ^***^P < 0.001.

The content of NO in the LPS group increased approximately 1.5 times when compared to the control group. At the same time, MG (80, 60, and 40 μM), HXRARG (80, 60, and 40 μM) and HXRA (8, 6, and 4 μM) also remarkably boosted the potential of RAW264.7 cells to produce NO in a dose-dependent manner. The generation of NO was comparable to that of the LPS group when the concentration of EG was 60 μM (Figure 4).

### Effects of EG, MG, HXRARG, and HXRA on iNOS, COX-2 Protein and mRNA Expression in RAW264.7 Cells

**Figure 5:**
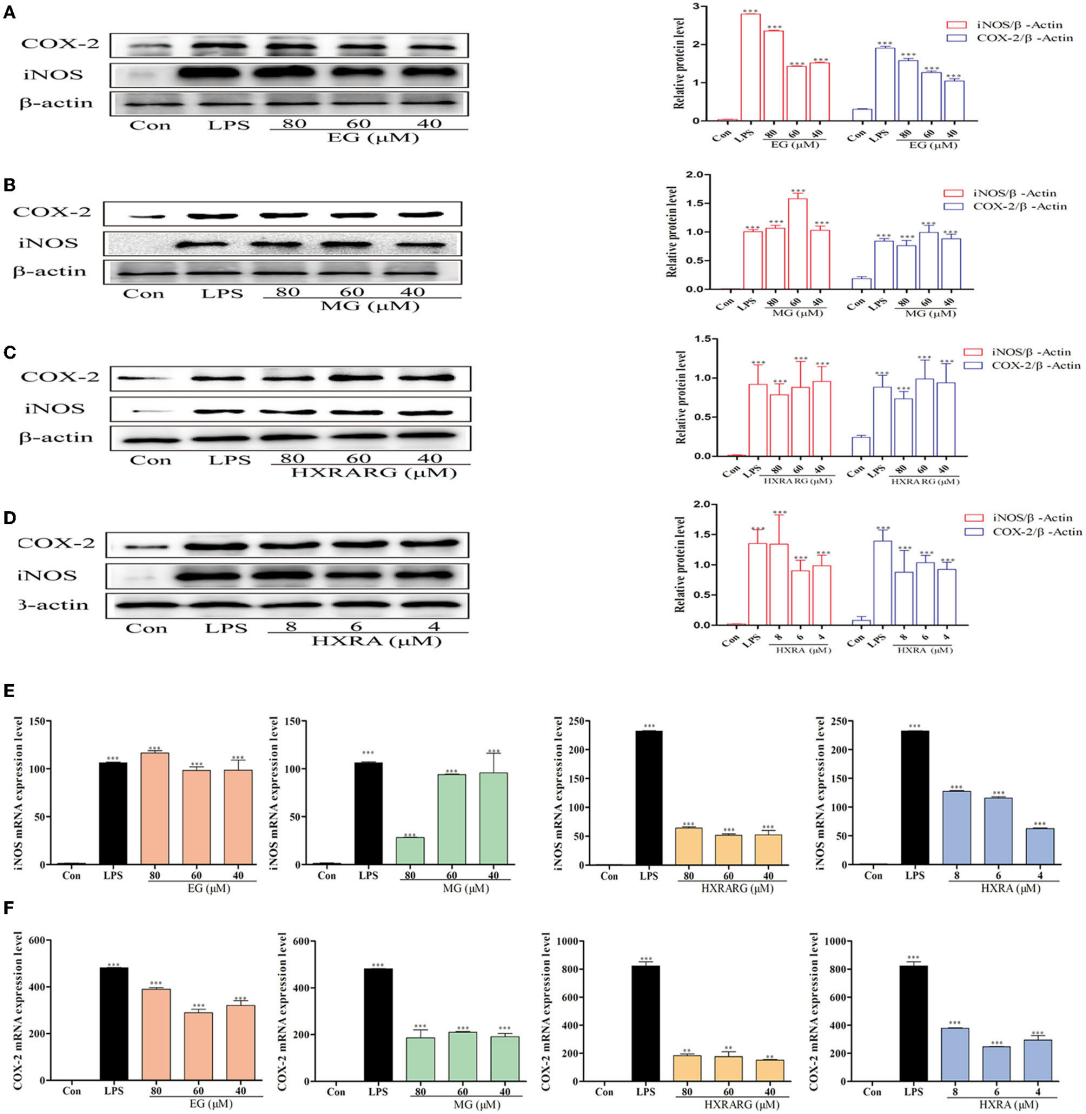
Effects of different concentrations of EG, MG, HXRARG, and HXRA on iNOS and Cox-2 protein **(A–D)** and mRNA **(E,F)** expression in RAW264.7 cells. **(A–D)**: the left side was the protein band diagram, and the right side was the protein quantification diagram. **(E)** mRNA expression of iNOS. **(F)** mRNA expression of COX-2. Data are expressed as the means ± SD. Compared with the control, ^**^P < 0.01, ^***^P < 0.001.

In RAW264.7 cells stimulated by LPS, the protein expression of iNOS and COX-2 was dramatically up-regulated (Figures 5A–D). When RAW264.7 cells were treated with compound for 24 h, the activities of total iNOS and Cox-2 were significantly increased when the expression of intracellular β-actin was almost unchanged. LPS greatly enhance the mRNA transcription of iNOS and COX-2, as seen in Figures 5E,F, and different concentrations of EG, MG, HXRARG or HXRA dramatically increase the transcription of COX-2 and iNOS mRNA in macrophages. In general, at the protein and gene levels, EG, MG, HXRARG, and HXRA can significantly enhance iNOS and COX-2 synthesis.

### Effects of EG, MG, HXRARG, and HXRA on the Expression of TNF-α, IL-6, and mRNA in RAW264.7 Cells

**Figure 6:**
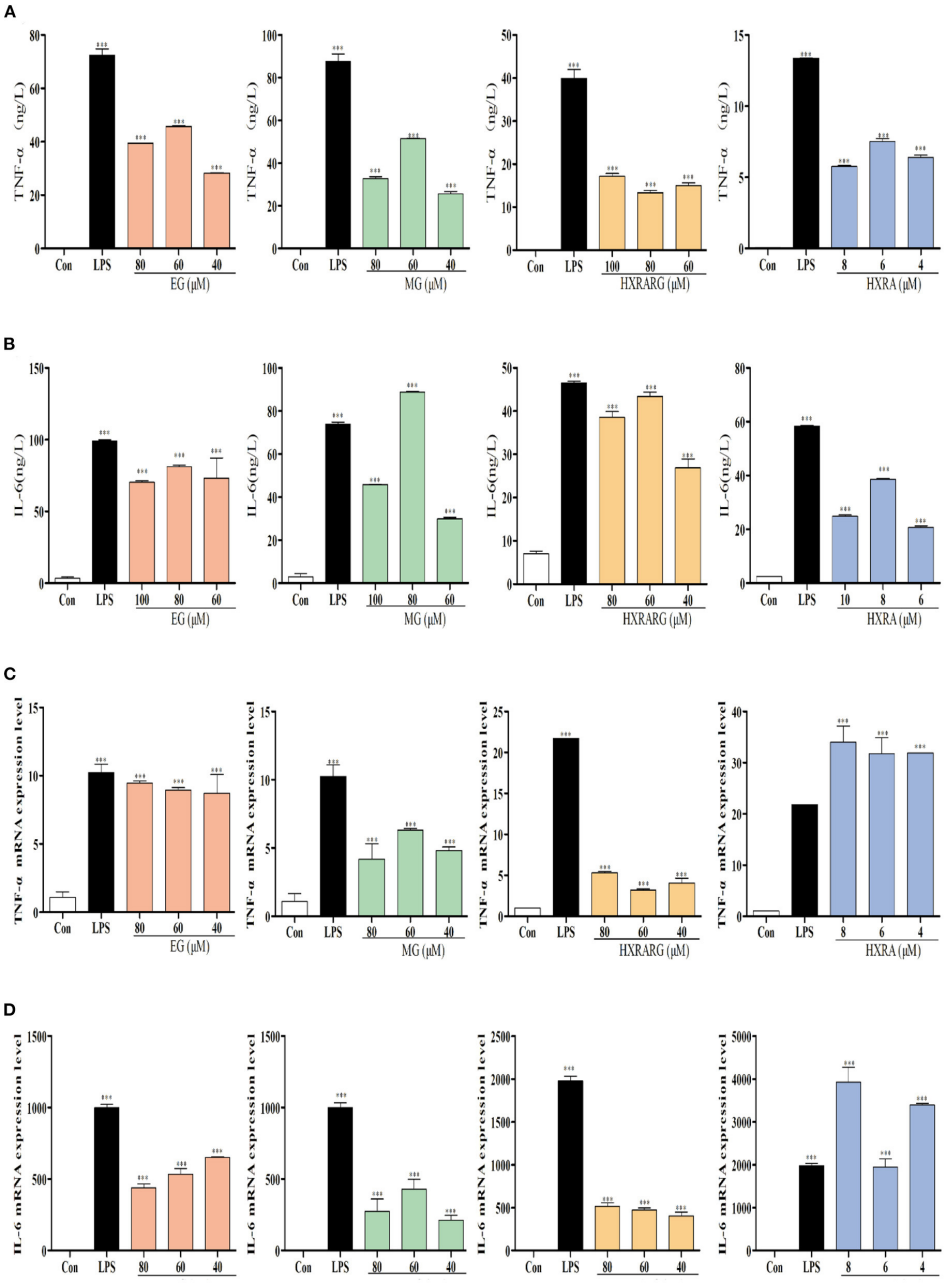
Effects of EG, MG, HXRARG and HXRA on the expression of tumor necrosis factor-α (TNF-α), interleukin-6 (IL-6) and mRNA in RAW264.7 cells. **(A)** secretion of IL-6 **(B)** secretion of TNF-α **(C)** TNF-α mRNA transcription **(D)** IL-6 mRNA transcription. Data are expressed as the means ± SD. Data are expressed as the means ±SD. Compared with the control, ^***^P < 0.001.

TNF-α and IL-6 secretion increased dramatically in RAW264.7 cells induced by LPS, as shown in Figure 6, and treatment of RAW264.7 cells with various concentrations of EG, MG, HXRARG, or HXRA for 24 h likewise enhance TNF-α secretion. In the Figures 6A,B, compared with the high and low dose groups, EG, MG, HXRARG, and HXRA could more effectively promote the secretion of IL-6 in the middle dose group. In Figures 6C,D, the mRNA expression of TNF-α and IL-6 increased significantly after LPS stimulation. TNF- and IL-6 mRNA expression was also considerably elevated after stimulation with various concentrations of EG, MG, HXRARG, or HXRA compared to the control group. These results suggested that EG, MG, HXRARG, or HXRA stimulate immune cells to secrete more TNF- α and IL-6 by promoting the mRNA translocation of TNF-α and IL-6.

### Effects of EG, MG, HXRARG, and HXRA on the Production of ROS in RAW264.7 Cells

**Figure 7:**
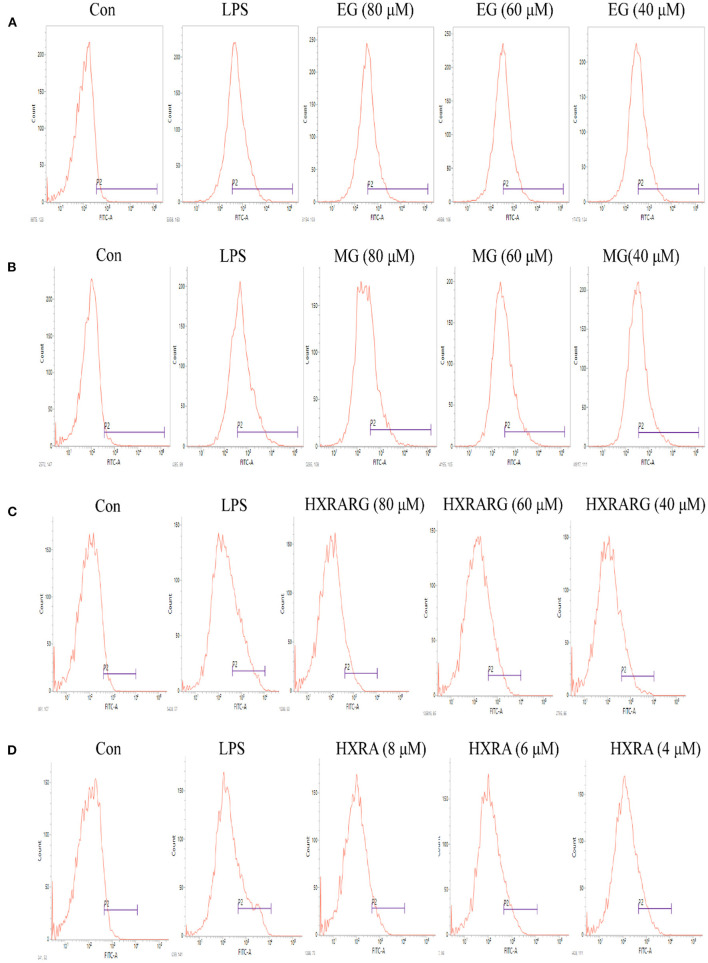
Effect of different concentrations of EG **(A)**, MG **(B)**, HXRARG **(C)**, and HXRA **(D)** on ROS production ability of RAW 264.7 cells.

The peak shape of the LPS group shifted to the right in comparison to the control group in Figure 7, suggesting that the ROS level of RAW264.7 cells increased following LPS stimulation. Compared with the control group, the peak shape also shifted to the right after stimulation with different concentrations of EG, MG, HXRARG, or HXRA, indicating that the production of ROS was significantly increased. Thus, we speculate that EG, MG, HXRARG, or HXRA enhance the immune function of macrophages in many ways.

### Effects of EG, MG, HXRARG, and HXRA on the Activation of NF-κB Pathways in RAW264.7 Cells

**Figure 8:**
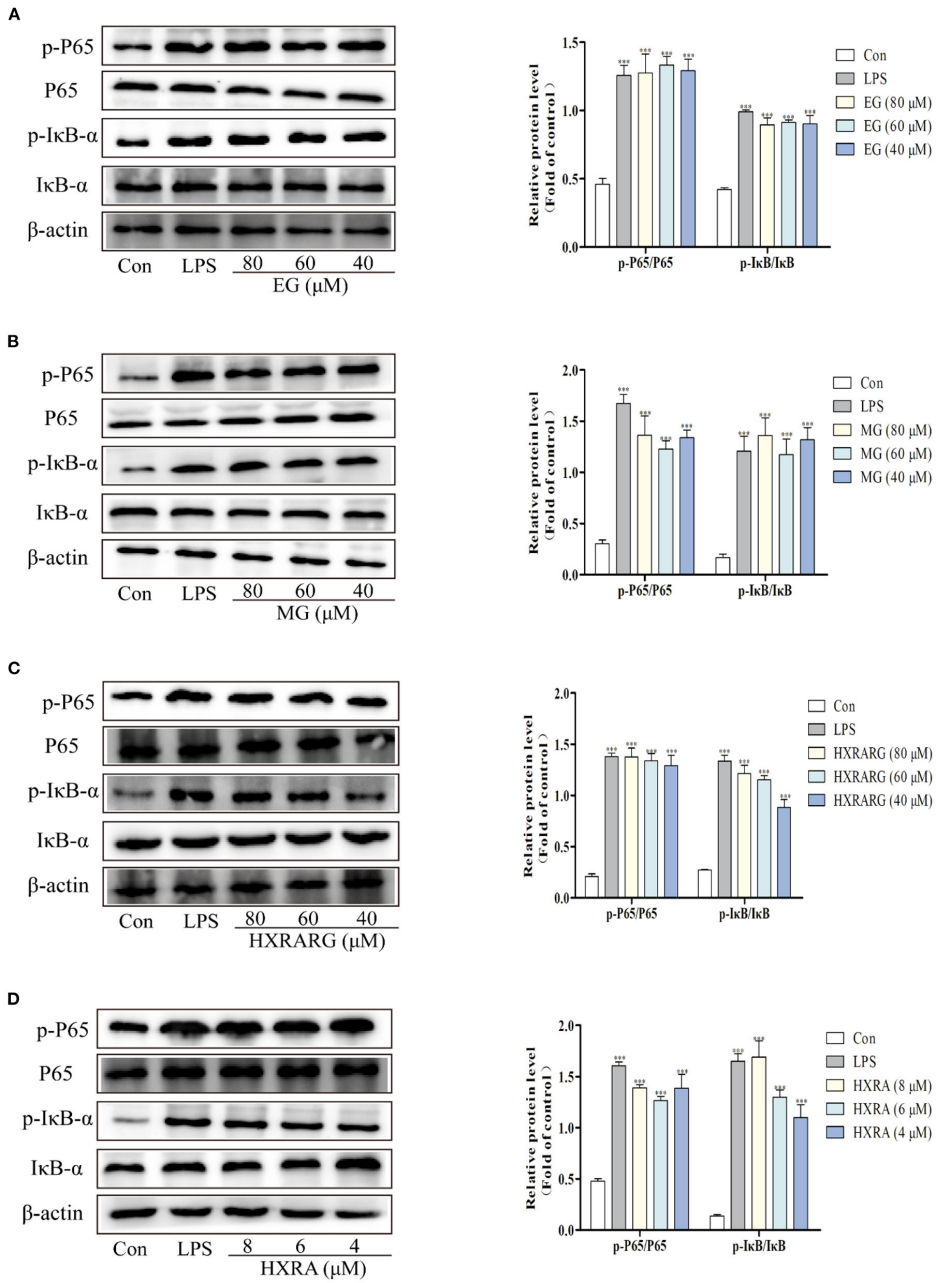
Effects of different concentrations of EG **(A)**, MG **(B)**, HXRARG **(C)**, and HXRA **(D)** on the expression of related proteins in NF-κB signaling pathway in RAW264.7 cells. The left side was the protein band diagram, and the right side was the protein quantification diagram. Data are expressed as the means ± SD. Compared with the control, ^***^P < 0.001.

The detection of NF-κB P65 in the nucleus and IκB in the cytoplasm is an important sign to judge whether the NF-κB pathway is activated or not. The expression levels of phosphorylated P65 and phosphorylated IκB in the LPS group were substantially higher than in the control group. After RAW264.7 cells were treated with EG, MG, HXRARG or HXRA, there was no significant effect on the expression of NF-κB P65 and IκB-α, but the phosphorylated expression of IκB-α and P65 was significantly increased, Figure 8 indicating that EG, MG, HXRARG, or HXRA activate NF-κB signal pathway so as to induce the activation of RAW264.7 cells.

### Effects of EG, MG, HXRARG, and HXRA on the Activation of MAPK Pathways in RAW264.7 Cells

**Figure 9:**
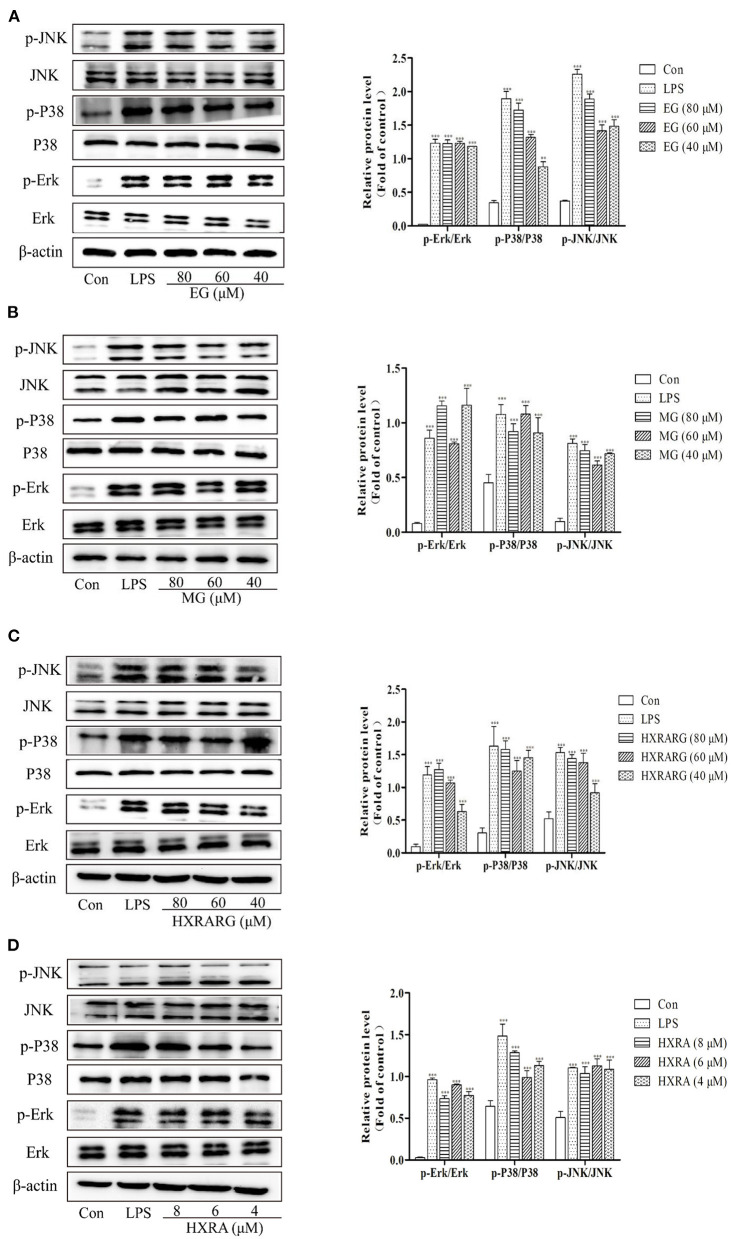
Effects of different concentrations of EG **(A)**, MG **(B)**, HXRARG **(C)**, and HXRA **(D)** on the expression of related proteins in MAPK signaling pathway in RAW264.7 cells. The left side was the protein band diagram, and the right side was the protein quantification diagram. Data are expressed as the means ± SD. Compared with the control, ^***^P < 0.001.

MAPK signal pathway is the convergence of many signal transduction pathways in cells. In addition, many bioactive components can regulate the production and secretion of NO and cytokines in macrophages by activating MAPK (36). In Figure 9, the LPS group had considerably higher phosphorylation levels of P38, ERK, and JNK than the control group. The ratios of p-P38/P38, p-Erk/Erk, and p-JNK/JNK were notably greater after stimulation with EG, MG, HXRARG, or HXRA than in the control group. These results suggest that EG, MG, HXRARG, or HXRA can activate MAPK signal pathway and induce immune response.

## Discussion

Plants have long been thought to be the foundation of nutrient-dense and useful diets, as well as the source of modern medicines throughout human history, which is not only because of their easy availability and low cost, but also because they are believed to have less harmful effects than synthetic drugs (37, 38). In addition, when used in combination with different conventional chemotherapy drugs, they produce synergistic effects, thereby reducing the dose of drugs used at the same time, optimizing efficacy and toxicity, and may overcome the problem of drug resistance (39). Therefore, it has a greater margin of safety and praiseworthy efficacy for a variety of diseases, and can be evaluated in clinical trials in a variety of cases (40, 41). However, it is estimated that only 15 percent of the 3,00,000 herbaceous species that exist in the world have been developed into pharmacological potential. Among several medicinal plants, *N. sativa* is regarded as one of the most valuable nutrient-dense herbs in human history, and numerous scientific investigations are underway to confirm its traditional use (42). At the same time, researchers found that *N. sativa* seeds can be utilized as nutritional supplements for COVID-19 patients as an adjuvant treatment, which set off an upsurge of research on *N. sativa* seeds (43–45).

Immunomodulatory effect is one of the most valuable characteristics of *N. sativa* seeds. In the last 20 years, significant progress has been made in the study of the health benefits of *N. sativa* seeds and its principal active ingredient, thymoquinone (TQ), on the immune system. TQ enhances immunomodulatory properties through T cells and NK cells (46). In most of the subjects who participated in the trial, after treatment with TQ, the ratio of CD4 and CD8 T cells increased by 55%, and the function of NK cells was improved, showing a significant effect (47). On BALB/c mice, Ghonime M investigated the therapeutic potential of a variety of medicinal herbs, including *N. sativa* seeds (48). Treated with various doses of *N. sativa* seed methanol extract (intraperitoneal injection), it was found that it could increase the total leukocyte count (up to 1.2 × 10^4^ cells/mm^3^). After administration of *N. sativa* seed extract, the activity of bone marrow cells also increased significantly. The spleen weight of *N. sativa* seed extract group increased significantly. Mice were immunosuppressed by cyclophosphamide, and the resistance of mice treated with *N. sativa* seed extract to lethal infection of *Candida albicans* was significantly recovered. The most of the studies on the immune activity of *N. sativa* seeds are based on animal models and are usually related to TQ, while there are few reports on the contribution of other active components to immune enhancement and action mechanism. In this study, RAW264.7 macrophages were used and cytotoxicity, phagocytosis, release of cytokines and effects of immune-related signaling pathways were used to evaluate the immune activity and immune mechanism of EG, MG, HXRA and HXRARG, which were isolated in *N. sativa* seeds.

The attention of people looking for new immune targets has shifted from extracellular to intracellular molecules with the in-depth study of cytokine transduction pathway (49). NF-κB is a ubiquitous and important nuclear transcription factor in the process of immunity. NF-κB interacts to IκBα in the cytoplasm in an inactive state under normal conditions (50–52). When the cell is activated, IκBα is ubiquitinated and then degraded, and then some specific sites of NF-κB subunits are phosphorylated to make NF-κB into an active form. Then, transfer to the nucleus and DNA binds to activate transcription, which participates in many biological processes such as apoptosis, cellular immune regulation, inflammation and tumorigenesis by regulating the expression of related chemokines, growth factors, COX-2 and nitric oxide synthase (53). P65 is the first NF-κB subunit found to be phosphorylated after the degradation of IκBα (54). The degradation of IκBα and the phosphorylation of p65 are important events in the activation of NF-κB signal pathway. The MAPK family contains three main kinases, namely Erk1/2, JNK and P38 MAPK. The co-activation of these kinases promotes cell proliferation, migration, invasion, angiogenesis, metastasis and apoptosis, which is very important for the development and activation of macrophages and T cells (55). The three kinases of MAPK are closely related but relatively independent, and are activated in the form of step-by-step phosphorylation.

**Figure 10:**
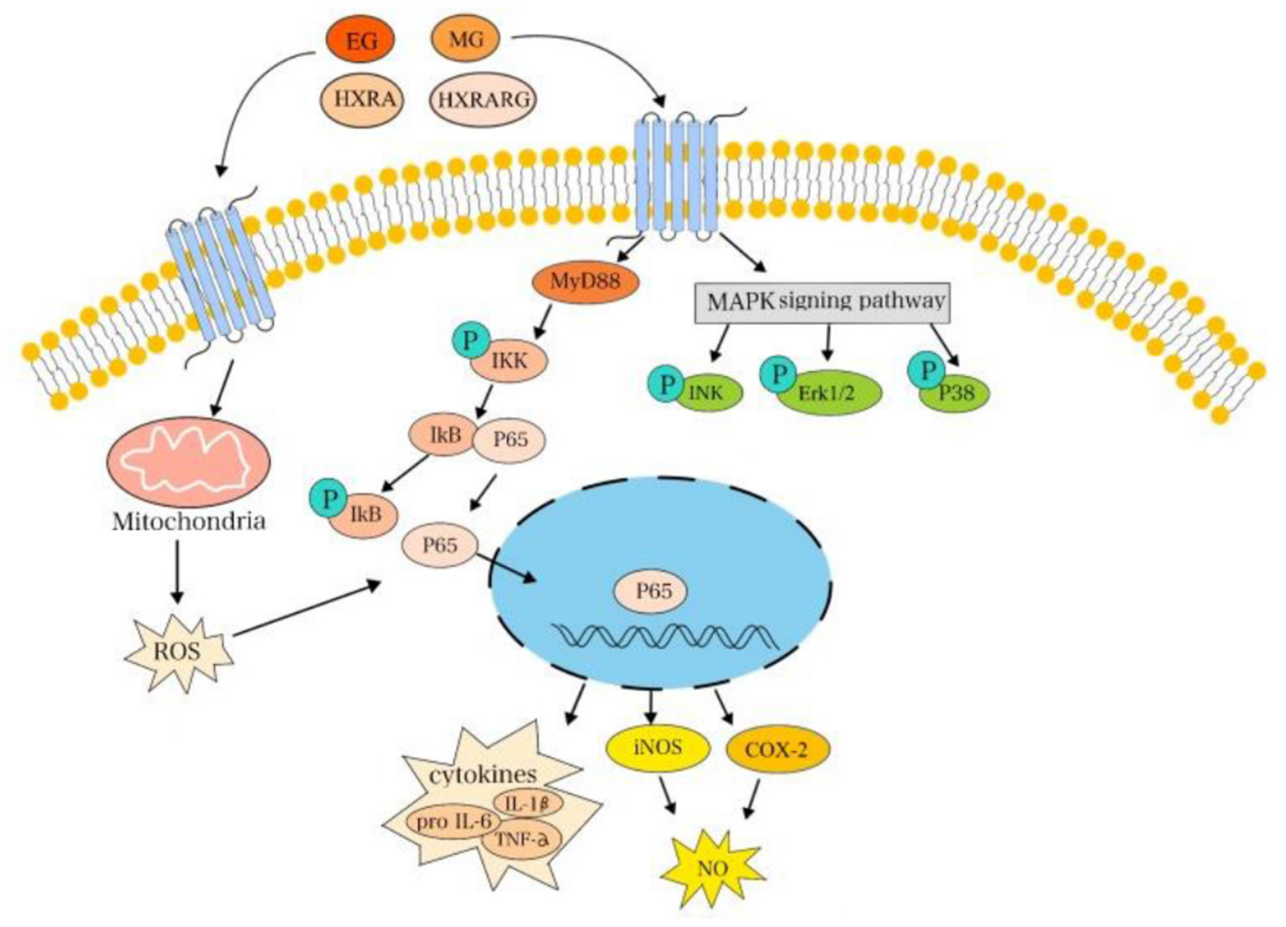
EG, MG, HXRARG, and HXRA activate NF-κB and MAPK signaling pathways to promote the activation of RAW264.7 cells.

The results showed that the safe doses of EG, MG, HXRARG and HXRA could activate the NF-κB pathway by promoting the phosphorylation of NF-κB p65 and IκBα in RAW264.7 cells, and activate the MAPK pathway by promoting the phosphorylation of JNK, Erk and P38, which leads to the changes of proliferation, phagocytosis and secretion of cytokines (Figure 10). He obtained two kinds of homogeneous polysaccharides PCSPA and PCSPB from *Crocus sativus*, and found that MAPK and NF-κB pathway can enhance cell phagocytosis, up-regulate the expression of surface molecules, promote the production of cytokines and chemokine and mRNA expression, and significantly activate RAW264.7 cells (56). Yan found that a new type of heterogalactan (WPEP-N-b) isolated from the *Pleurotus eryngi* fruiting body activates macrophages by increasing the production and phagocytosis of NO, TNF-α, IL-6, and IL-1β. At the molecular level, WPEP-N-b activates macrophages through TLR2-mediated MAPK and NF-κB signaling pathways (57). El-Obeid reported that melanin extracted from *N. sativa* seeds can enhance the mRNA expression of TNF-α, IL-6 and vascular endothelial growth factor (VEGF) in monocytes, peripheral blood mono-nuclear cells (PBMC) and THP-1 cells (58), which is consistent with our results.

The medicinal and health promotion characteristics of plant products are related to the existence of phytochemical components such as some secondary metabolites, phenolic compounds and terpenes in natural essential oils, which show many positive biological activities (59). *N. sativa* is a good source of essential nutrients, and supplementation of *N. sativa* can improve the overall antioxidant status of the body. Immune enhancement and immunomodulatory effects may be due to its interference with the ability of immunomodulators (60).

EG and MG, as monosaccharide derivatives isolated from *N. sativa* seeds, showed good potential to regulate and enhance the immune system. Huang found that monosaccharide derivative GPP could activate RAW264.7 cells by increasing phagocytosis, NO production and mRNA expression of iNOS and TNF-α (61, 62). Although the mechanism of immune enhancement of GPP was not studied *in vitro*, GPP could significantly improve the activity of natural killer (NK) cells and macrophage phagocytosis in mice after oral administration of 0, 3.6, 120, and 360 mg/kg body weight (mg/kg BW) for 30 days. Tsvetkov YE's immunobiological evaluation of chitin and chitosan-derived oligosaccharides on RAW264.7 cells *in vitro* showed that the beneficial immunomodulatory effects were related to cell proliferation, phagocytosis, cytokine release and cellular respiratory burst, which was consistent with the results of this experiment (63). Levine R and other studies have found that arabinoxylan with immunomodulatory effect can be regarded as an effective bioactive food supplement related to many health improvement functions (64).

Triterpenoid saponins have a variety of pharmacological effects, including hypoglycemia, anticancer, neuroprotection, liver protection, inhibition of lipid production, antibacterial, antioxidation, anti-angiogenesis and so on (65, 66). However, most of the studies on triterpenoid saponins are focused on anti-inflammatory effect, and there are few studies on immune effect. The anti-inflammatory activity of ginsenoside PG-RT_8_ was evaluated by RAW264.7 macrophages and THP-1 cells stimulated by LPS (67). It was found that PG-RT_8_ could attenuate the release of LPS-mediated pro-inflammatory factors (IL-1b, IL-6, iNOS, COX2 and MMP-9) on a dose-dependent manner. In addition, PG-RT_8_ strongly inhibited the production of ROS and NO. Salidroside derivative (PTs29) significantly inhibited LPS-induced secretion of pro-inflammatory factors TNF-α and IL-6 in THP-1 cells by stimulating the phosphorylation of AMPK and acetyl-CoA carboxylase (ACC). It indicated that triterpene saponins have good immune enhancement effect (68).

It is very important to improve personal immunity by eating nutritionally balanced food. So, balanced food with therapeutic functions is very beneficial. *N. sativa* seeds are very safe and effective as immune supplements. Ali and Blunden proved that *N. sativa* seed extracts or oils did not have significant toxic or adverse effects on liver or kidney function (69). In addition, monosaccharide derivatives or saponins derivatives are usually low in our diets, which is an important contribution to some dietary habits. Our investigation also proved that *N. sativa* seed contains the active components to improve immunity and it will be the beneficial supplements for immunodeficient human.

## Conclusion

Four compounds, Ethyl-α-D-galactopyranoside (EG), Methyl-α-D-glucoside (MG), 3-*O*-[β-*D*-xylopyranose-(1 → 3)-α-*L*-rhamnose-(1 → 2)-α-*L*-arabinose]-28-*O*-[α-*L*-rhamnose-(1 → 4)-β-*D*-glucopyranose-L-(1 → 6)-β-*D*-glucopyranose]-hederagenin (HXRARG) and 3-*O*-[β-*D*-xylopyranose-(1 → 3)-α-*L*-rhamnose-(1 → 2)-α-*L*-arabinose]-hederagenin (HXRA), were isolate and identified in *N. sativa* seeds. They could significantly increase the production of NO, COX-2, TNF-α, and IL-6 by activating the JNK, ERK1/2, P38, and NF-κB pathways. Therefore, *N. sativa* seeds exhibited immune-enhancing potential, and might be developed as an immunopotentiator in the near future.

## Data Availability Statement

The original contributions presented in the study are included in the article/Supplementary Material, further inquiries can be directed to the corresponding author/s.

## Author Contributions

JW designed the experiments and wrote the manuscript. BW performed experiments. QW analyzed and summarized data. YC was responsible for formal analysis and visualization. AA contributed to the data acquisition. YZ supervised project administration and critically reviewed the manuscript. WK provided resources, funding, and reviewed manuscript. All authors contributed to the article and approved the submitted version.

## Funding

This work was funded by Research on Precision Nutrition and Health Food, Department of Science and Technology of Henan Province (CXJD2021006).

## Conflict of Interest

The authors declare that the research was conducted in the absence of any commercial or financial relationships that could be construed as a potential conflict of interest.

## Publisher's Note

All claims expressed in this article are solely those of the authors and do not necessarily represent those of their affiliated organizations, or those of the publisher, the editors and the reviewers. Any product that may be evaluated in this article, or claim that may be made by its manufacturer, is not guaranteed or endorsed by the publisher.
