# Supplementary figures and images for: The Immunomodulatory Effects of Active Ingredients From Nigella sativa in RAW264.7 Cells Through NF-κB/MAPK Signaling Pathways
Source: Front Nutr. 2022 May 31;9:899797. doi: 10.3389/fnut.2022.899797 (PMC9194833; doi:10.3389/fnut.2022.899797)

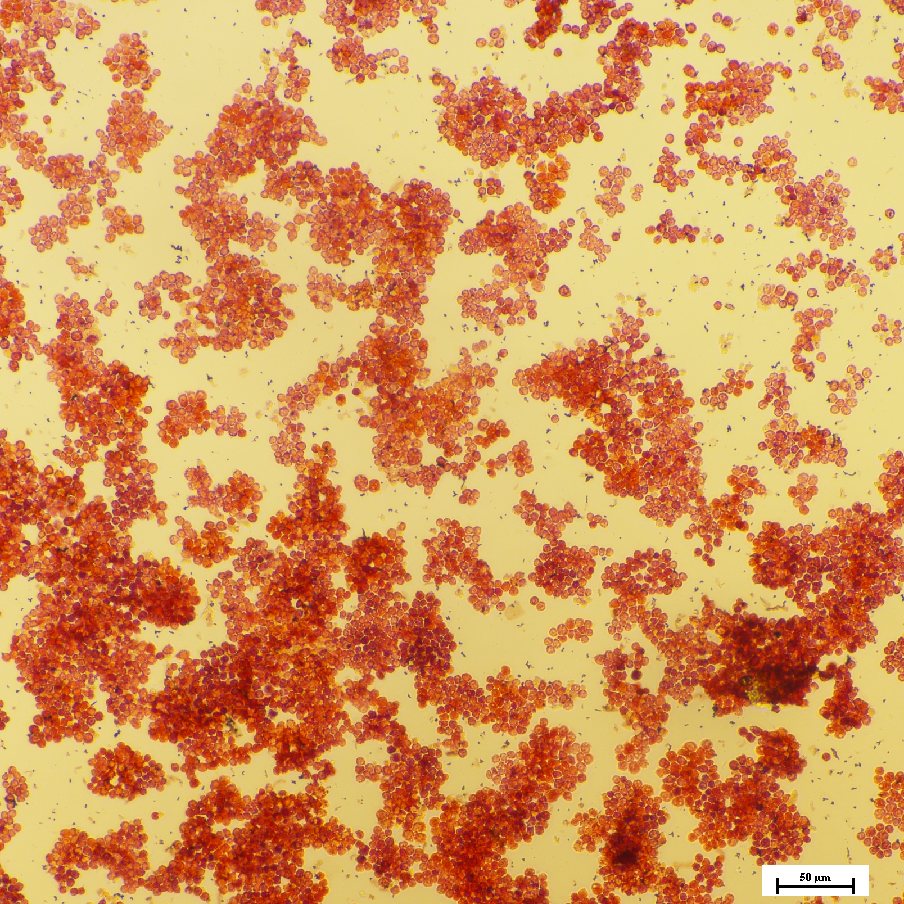

Supplement: Supplementary file 1 [file Data_Sheet_1.ZIP › EG and MG of Image taken with electron microscope/Con-.tif]

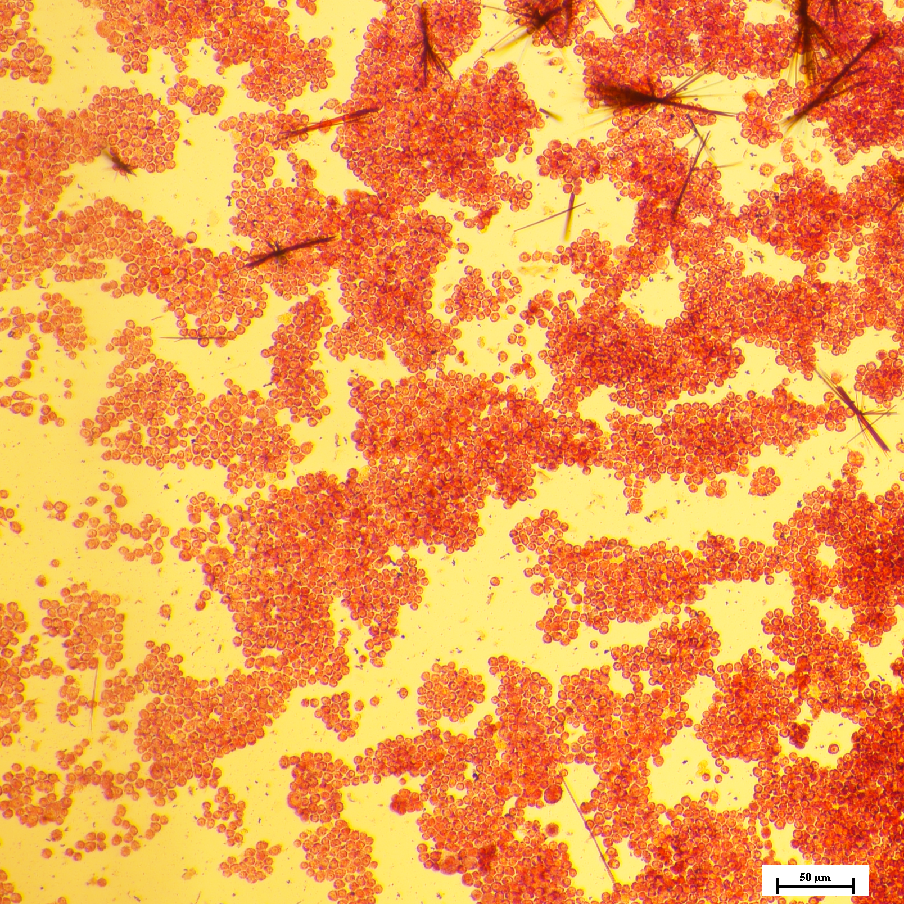

Supplement: Supplementary file 1 [file Data_Sheet_1.ZIP › EG and MG of Image taken with electron microscope/EG(40 a╠M).tif]

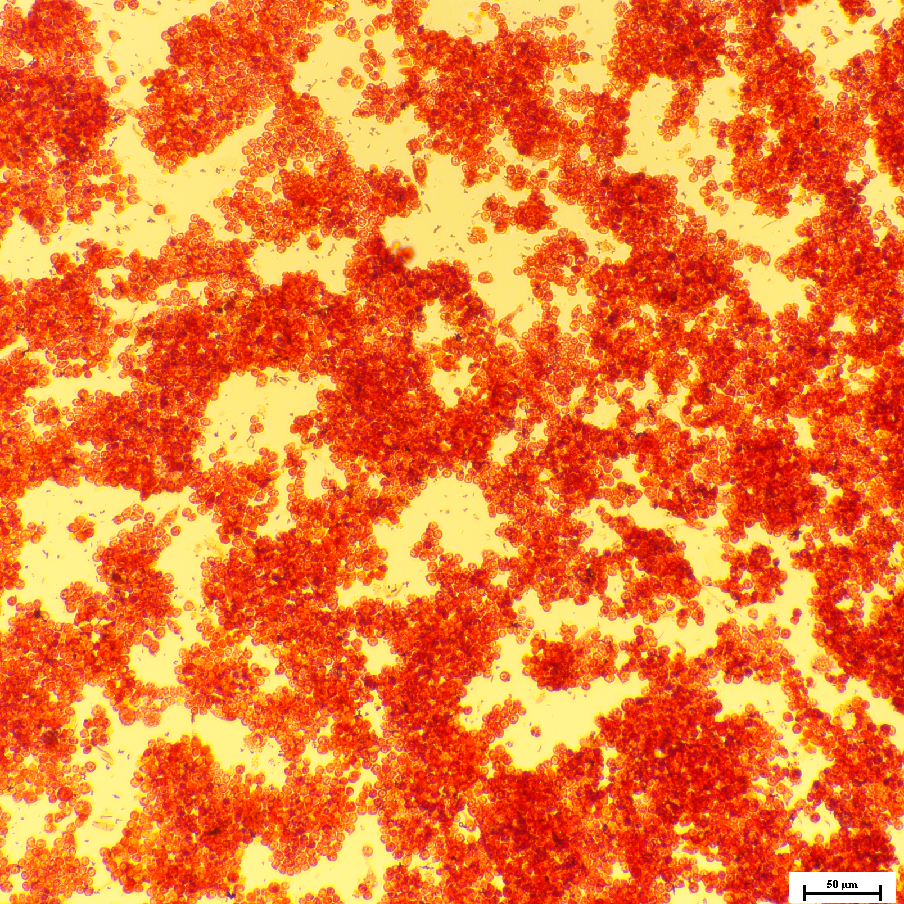

Supplement: Supplementary file 1 [file Data_Sheet_1.ZIP › EG and MG of Image taken with electron microscope/EG(60 a╠M).tif]

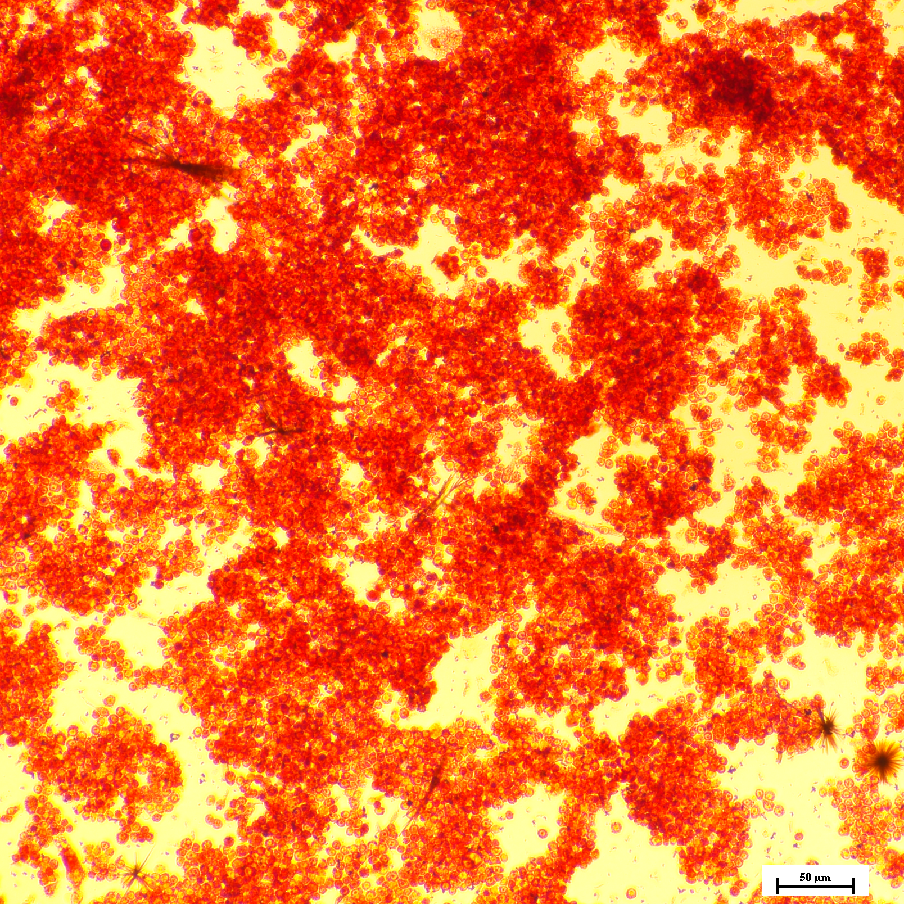

Supplement: Supplementary file 1 [file Data_Sheet_1.ZIP › EG and MG of Image taken with electron microscope/EG(80 a╠M).tif]

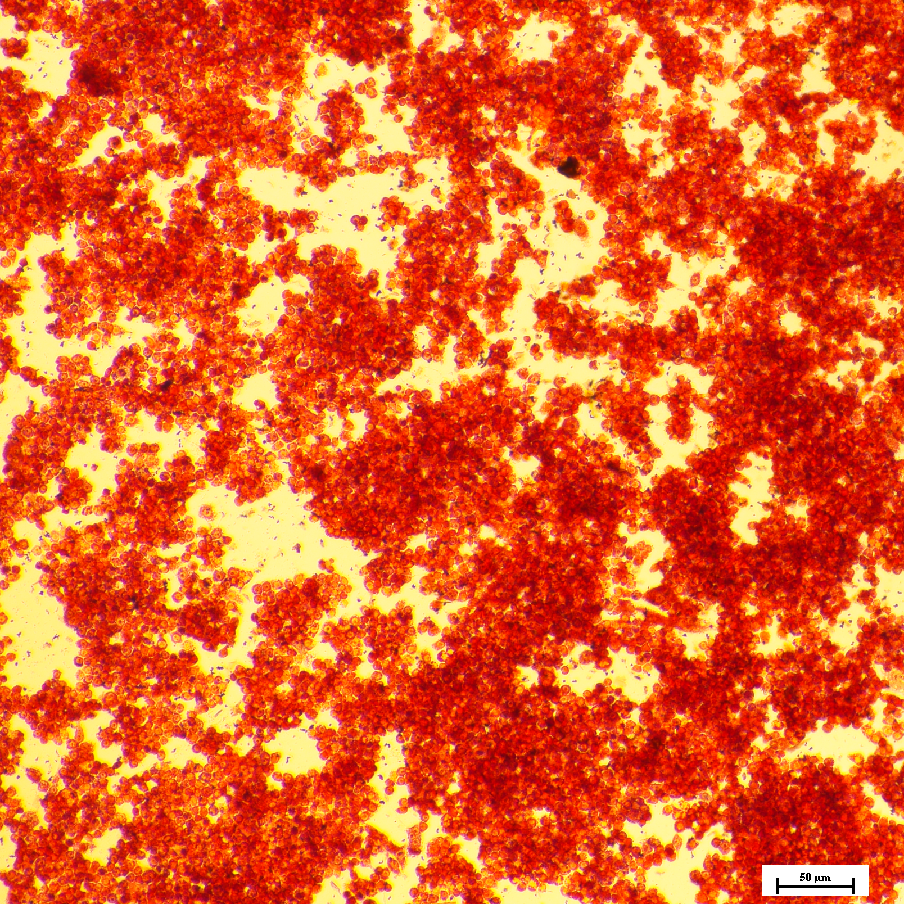

Supplement: Supplementary file 1 [file Data_Sheet_1.ZIP › EG and MG of Image taken with electron microscope/LPS-.tif]

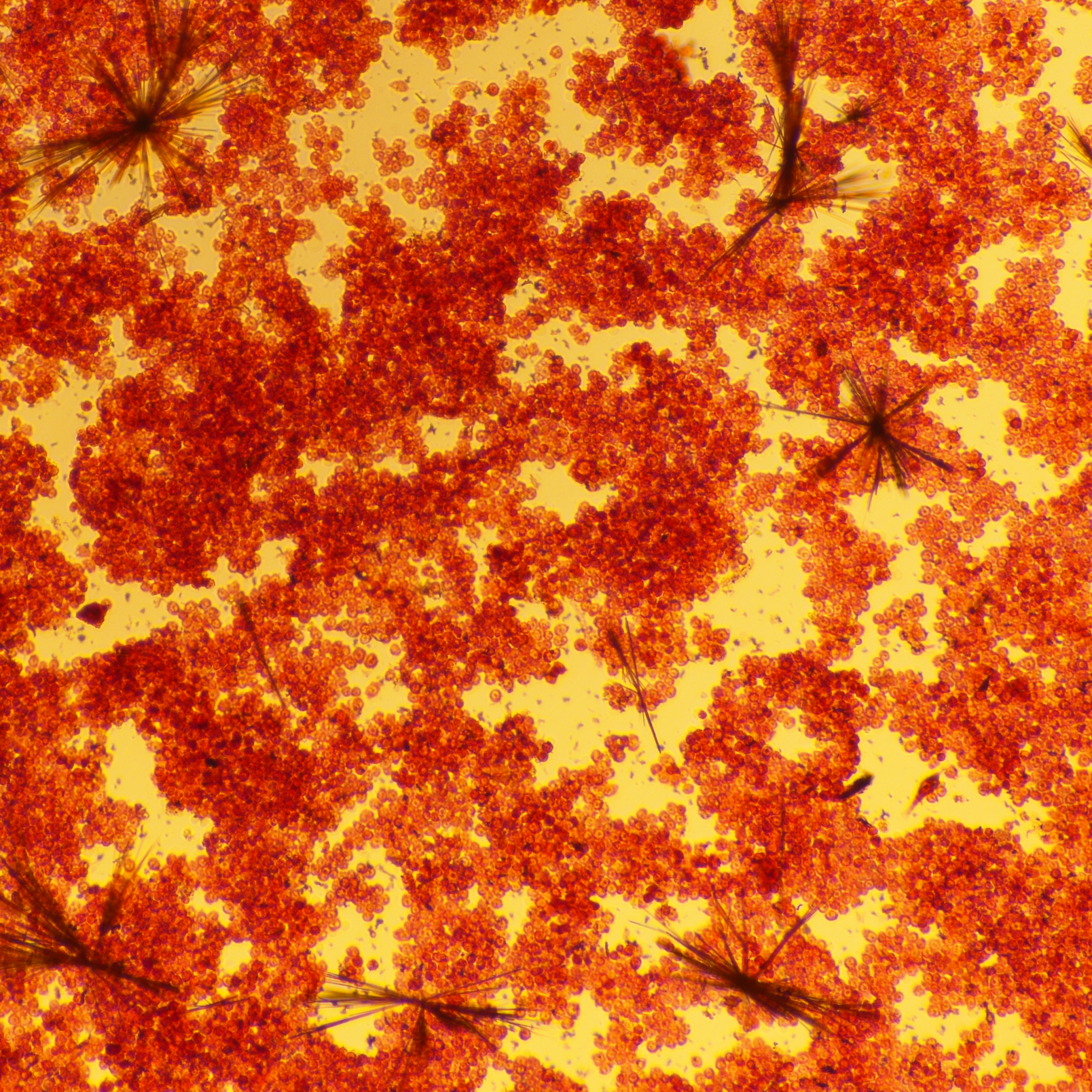

Supplement: Supplementary file 1 [file Data_Sheet_1.ZIP › EG and MG of Image taken with electron microscope/MG(40 a╠M).tif]

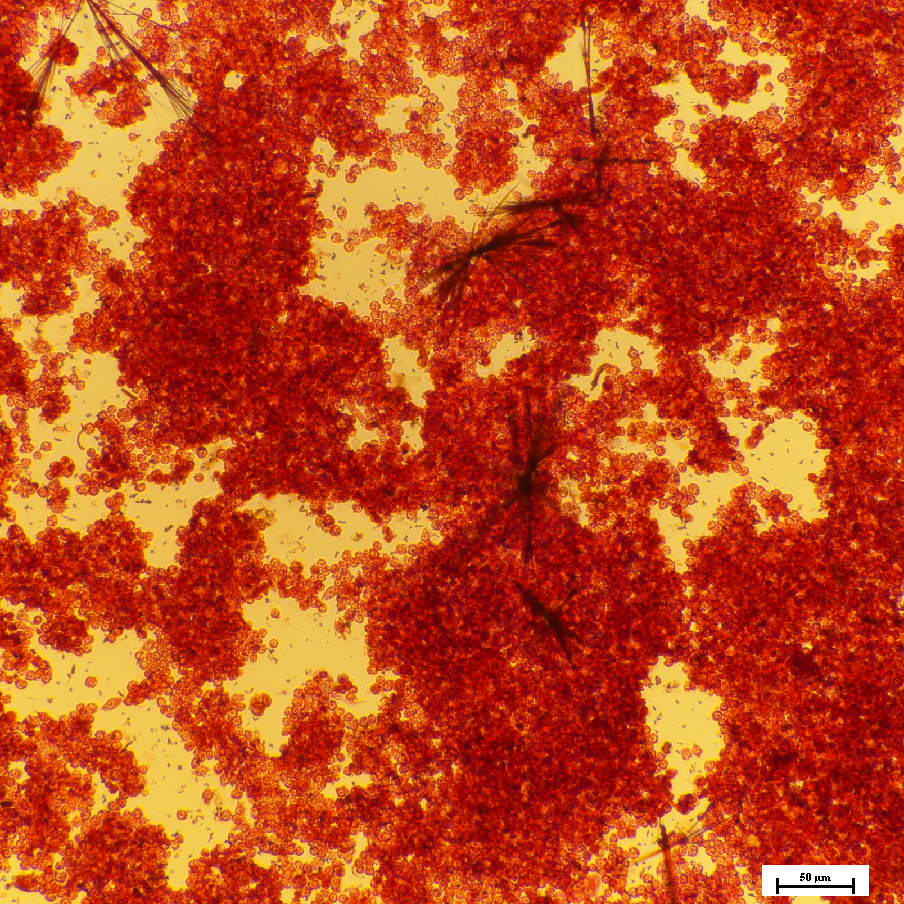

Supplement: Supplementary file 1 [file Data_Sheet_1.ZIP › EG and MG of Image taken with electron microscope/MG(60 a╠M).tif]

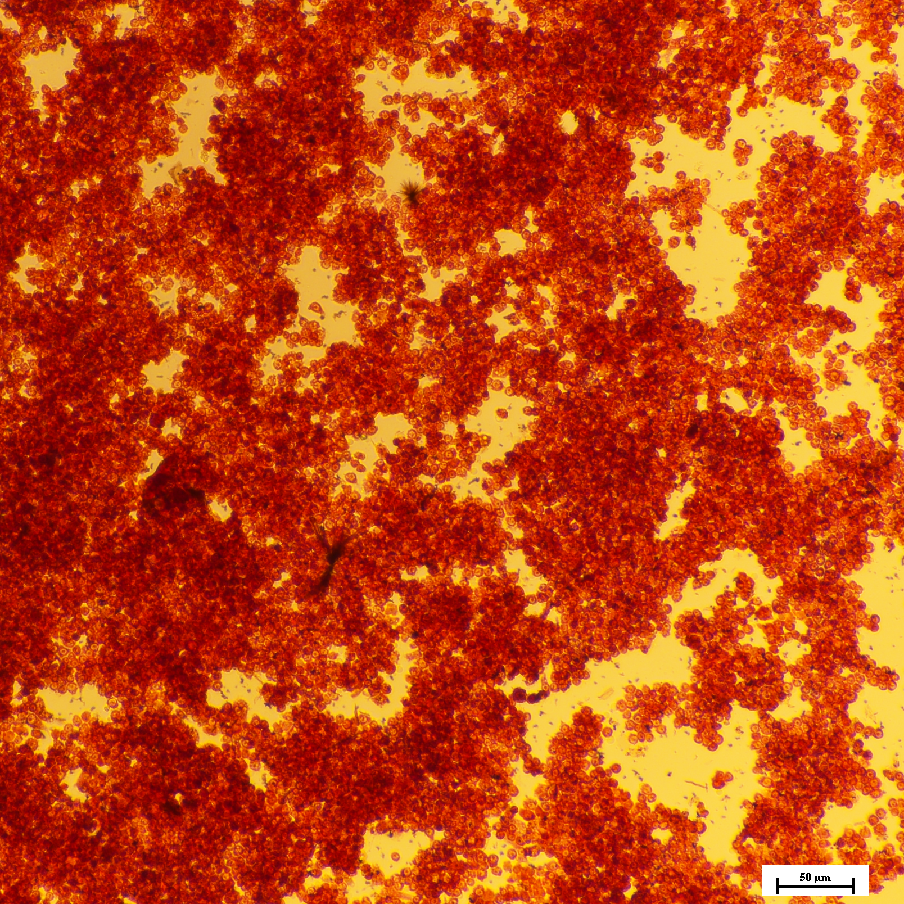

Supplement: Supplementary file 1 [file Data_Sheet_1.ZIP › EG and MG of Image taken with electron microscope/MG(80 a╠M).tif]

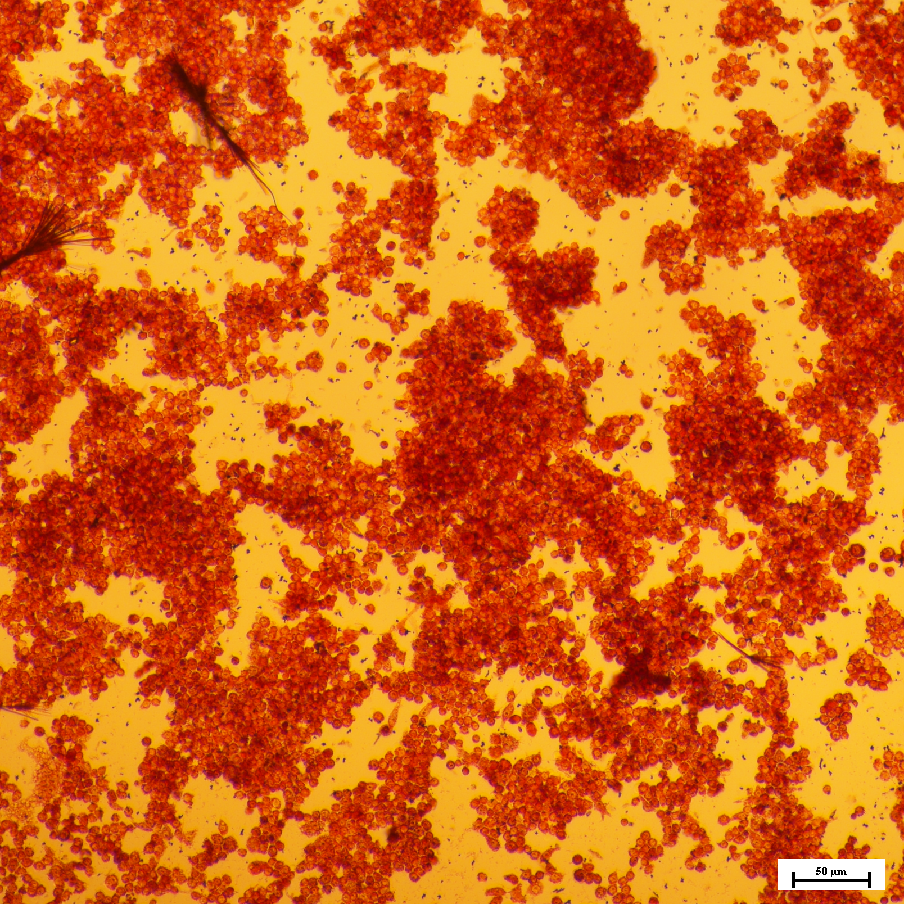

Supplement: Supplementary file 2 [file Data_Sheet_2.ZIP › HXRA and HXRARG of Image taken with electron microscope/HXRA (4 a╠M).tif]

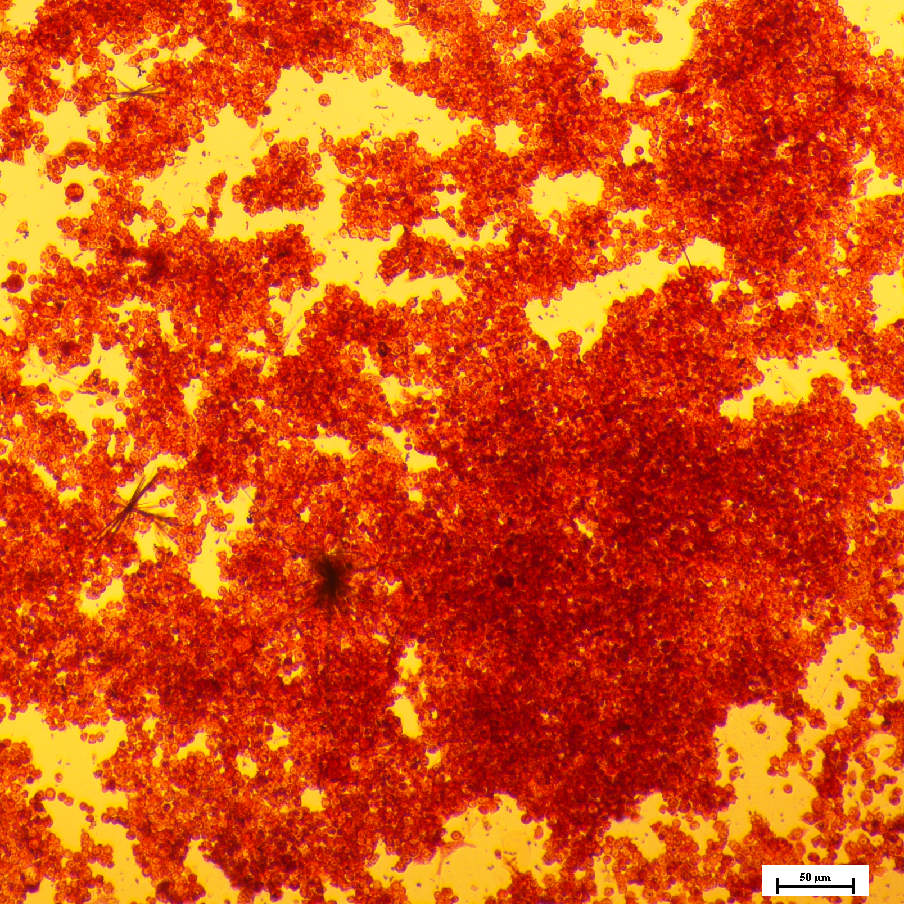

Supplement: Supplementary file 2 [file Data_Sheet_2.ZIP › HXRA and HXRARG of Image taken with electron microscope/HXRA (6 a╠M).tif]

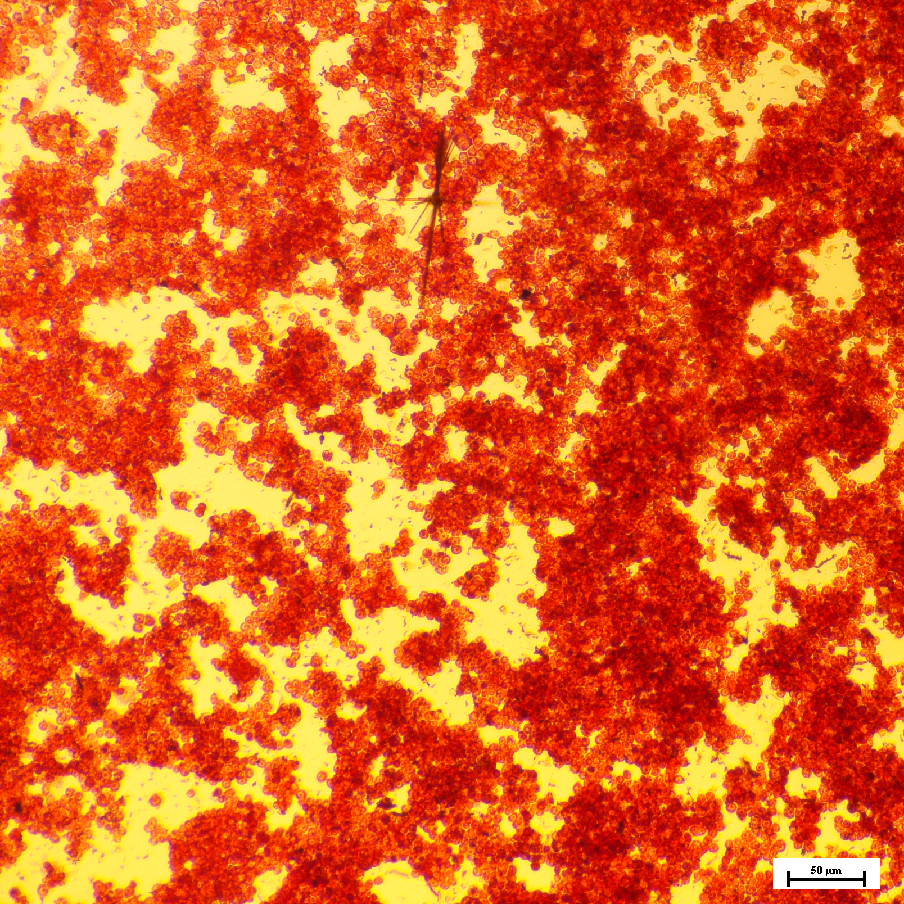

Supplement: Supplementary file 2 [file Data_Sheet_2.ZIP › HXRA and HXRARG of Image taken with electron microscope/HXRA (8 a╠M).tif]

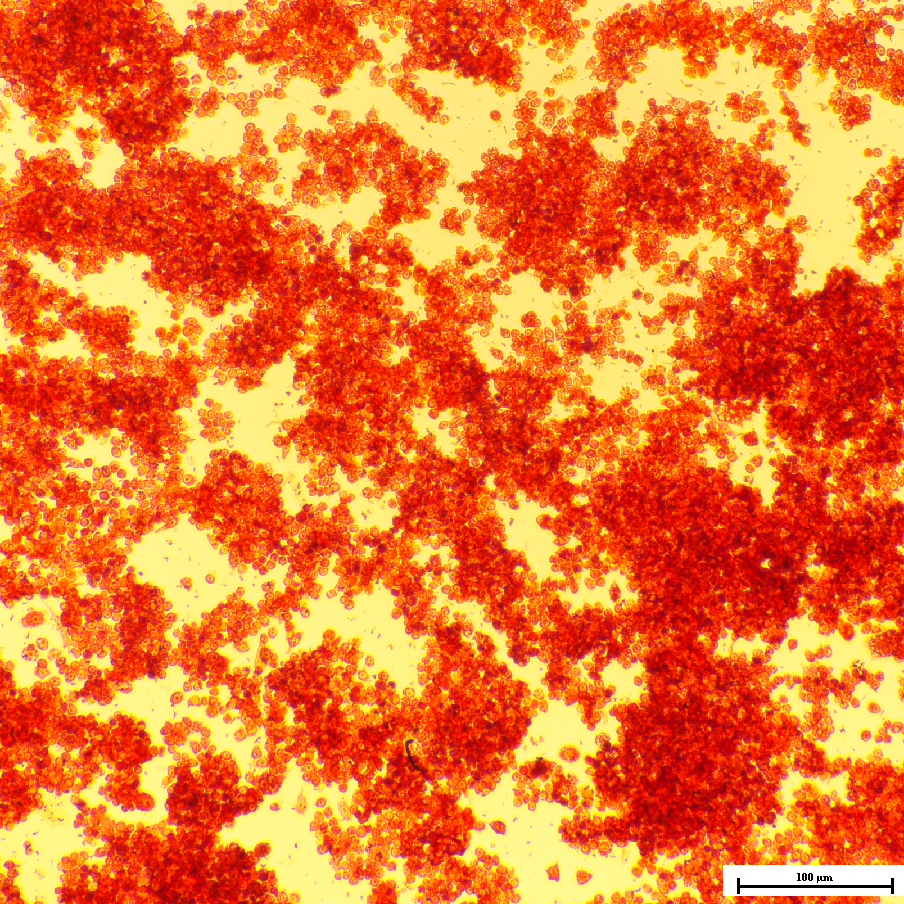

Supplement: Supplementary file 2 [file Data_Sheet_2.ZIP › HXRA and HXRARG of Image taken with electron microscope/HXRARG (40 a╠M).tif]

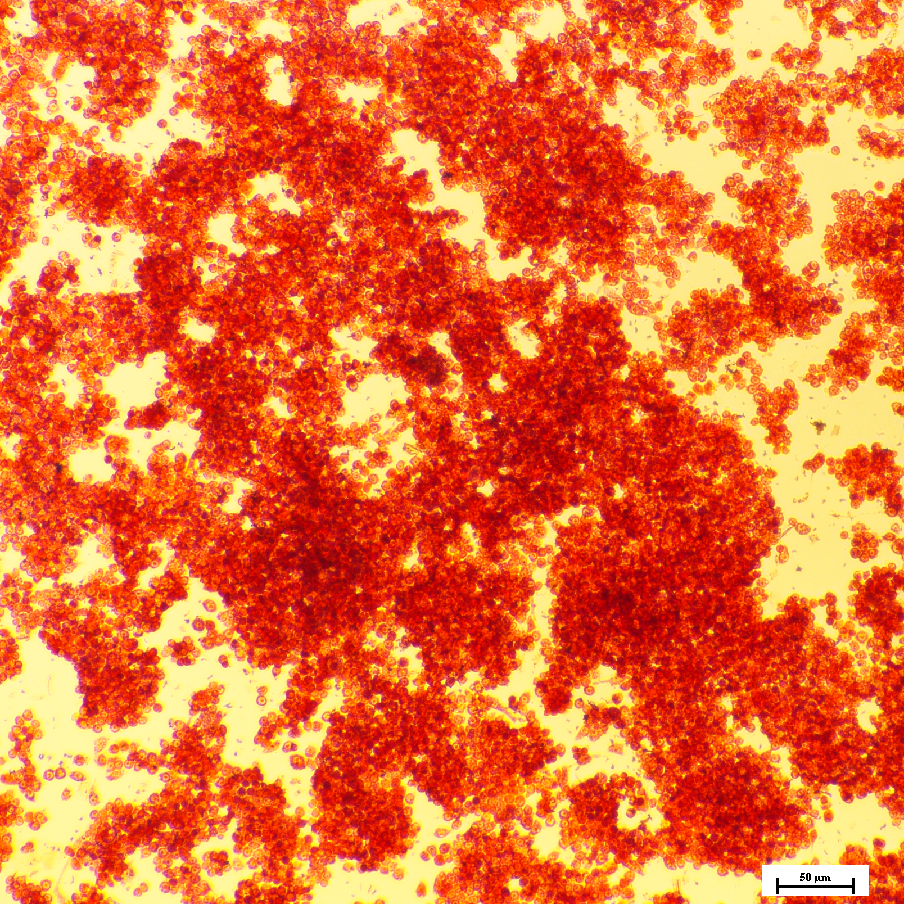

Supplement: Supplementary file 2 [file Data_Sheet_2.ZIP › HXRA and HXRARG of Image taken with electron microscope/HXRARG (60 a╠M).tif]

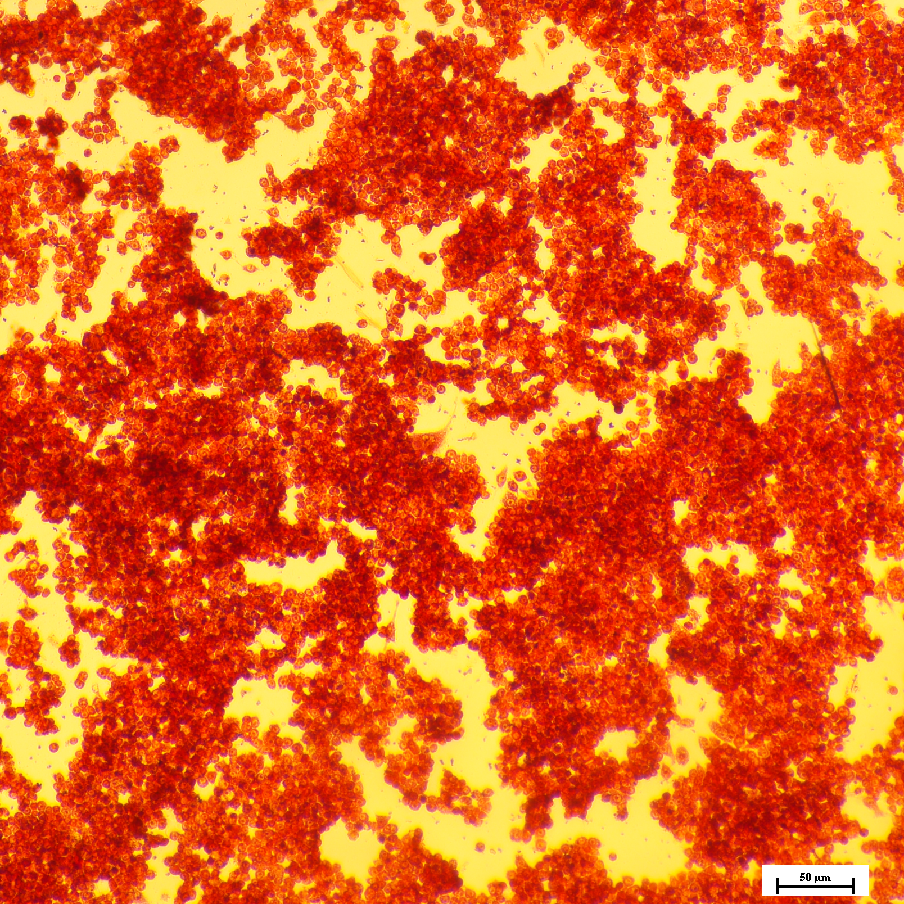

Supplement: Supplementary file 2 [file Data_Sheet_2.ZIP › HXRA and HXRARG of Image taken with electron microscope/HXRARG (80 a╠M).tif]

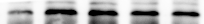

Supplement: Supplementary file 3 [file Data_Sheet_3.ZIP › Original Data/Fig. 5/INOS and Cox-2 protein expression/EG-Cox2.tif]

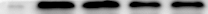

Supplement: Supplementary file 3 [file Data_Sheet_3.ZIP › Original Data/Fig. 5/INOS and Cox-2 protein expression/EG-INOS.tif]

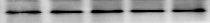

Supplement: Supplementary file 3 [file Data_Sheet_3.ZIP › Original Data/Fig. 5/INOS and Cox-2 protein expression/EG-a┬-antin.tif]

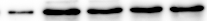

Supplement: Supplementary file 3 [file Data_Sheet_3.ZIP › Original Data/Fig. 5/INOS and Cox-2 protein expression/HXRA -Cox2.tif]

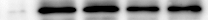

Supplement: Supplementary file 3 [file Data_Sheet_3.ZIP › Original Data/Fig. 5/INOS and Cox-2 protein expression/HXRA -INOS.tif]

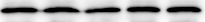

Supplement: Supplementary file 3 [file Data_Sheet_3.ZIP › Original Data/Fig. 5/INOS and Cox-2 protein expression/HXRA -a┬-actin.tif]

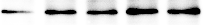

Supplement: Supplementary file 3 [file Data_Sheet_3.ZIP › Original Data/Fig. 5/INOS and Cox-2 protein expression/HXRARG -Cox2.tif]

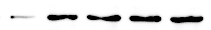

Supplement: Supplementary file 3 [file Data_Sheet_3.ZIP › Original Data/Fig. 5/INOS and Cox-2 protein expression/HXRARG -INOS.tif]

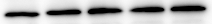

Supplement: Supplementary file 3 [file Data_Sheet_3.ZIP › Original Data/Fig. 5/INOS and Cox-2 protein expression/HXRARG -a┬-actin.tif]

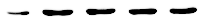

Supplement: Supplementary file 3 [file Data_Sheet_3.ZIP › Original Data/Fig. 5/INOS and Cox-2 protein expression/MG-Cox2.tif]

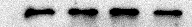

Supplement: Supplementary file 3 [file Data_Sheet_3.ZIP › Original Data/Fig. 5/INOS and Cox-2 protein expression/MG-INOS.tif]

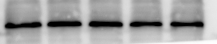

Supplement: Supplementary file 3 [file Data_Sheet_3.ZIP › Original Data/Fig. 5/INOS and Cox-2 protein expression/MG-a┬-actin.tif]

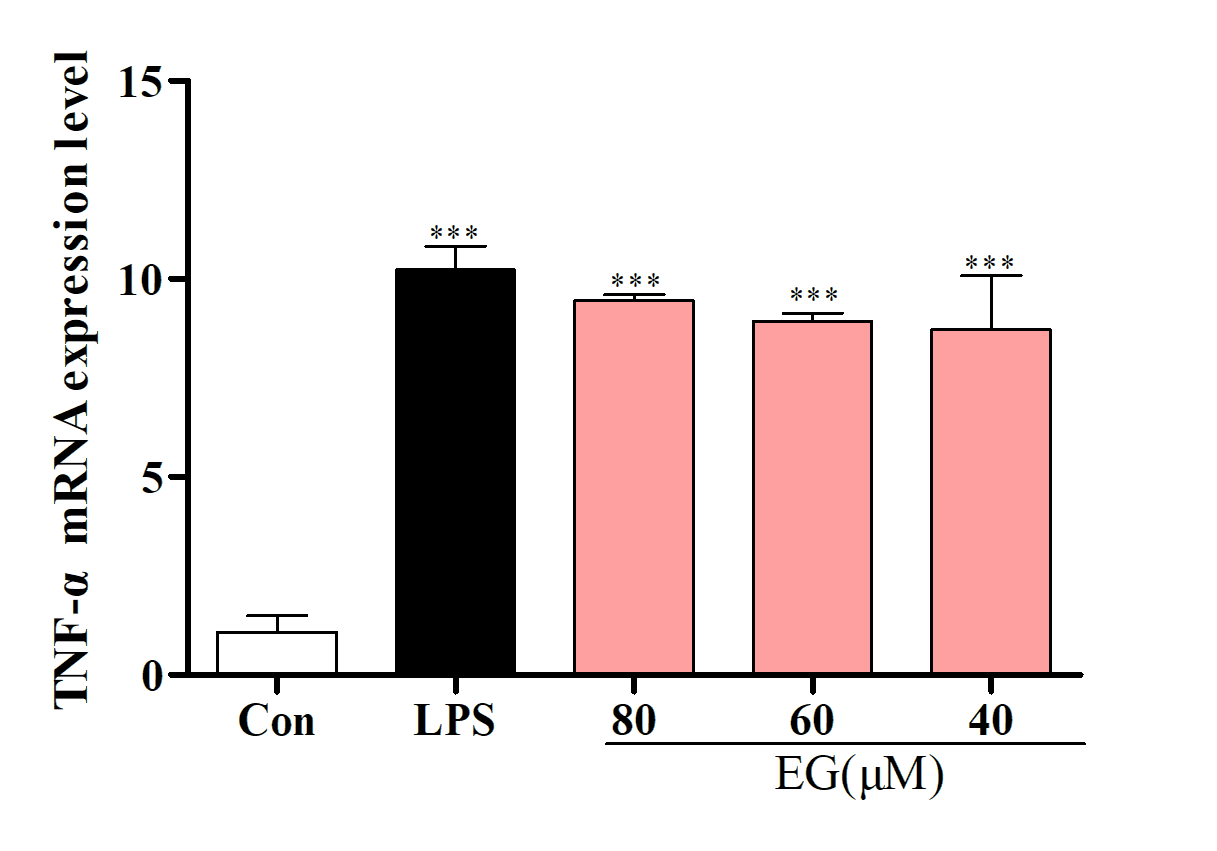

Supplement: Supplementary file 3 [file Data_Sheet_3.ZIP › Original Data/Fig. 6/TNF-a┴ mRNA/EG-TNF.tif]

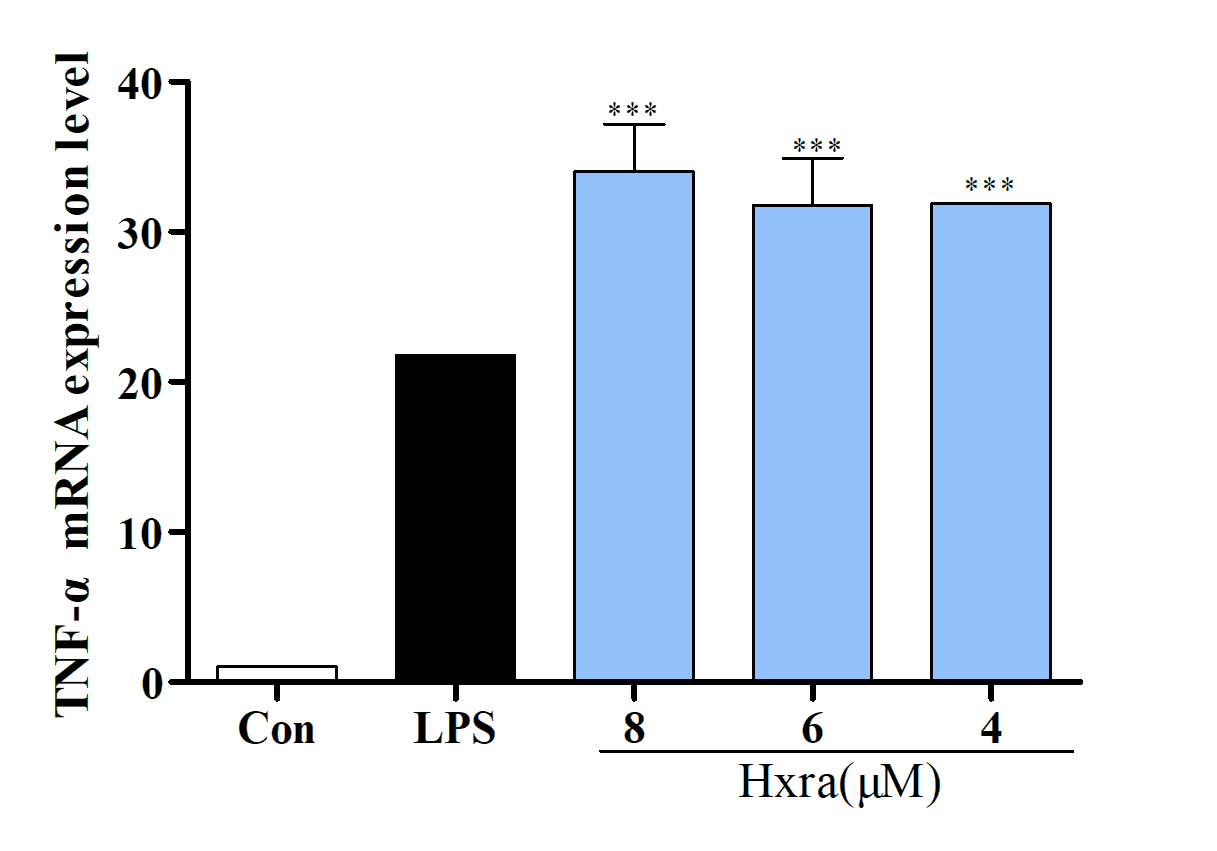

Supplement: Supplementary file 3 [file Data_Sheet_3.ZIP › Original Data/Fig. 6/TNF-a┴ mRNA/Hxra-TNF.tif]

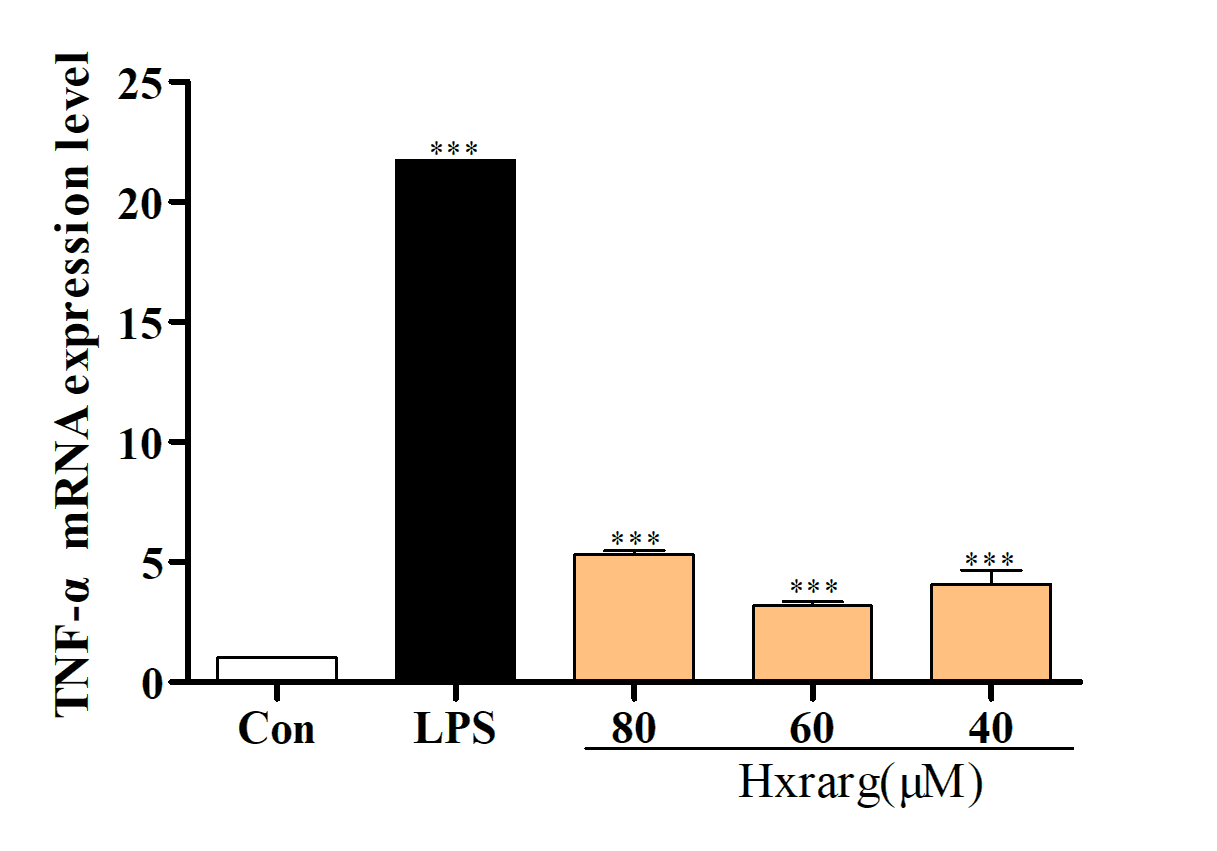

Supplement: Supplementary file 3 [file Data_Sheet_3.ZIP › Original Data/Fig. 6/TNF-a┴ mRNA/Hxrarg-TNF.tif]

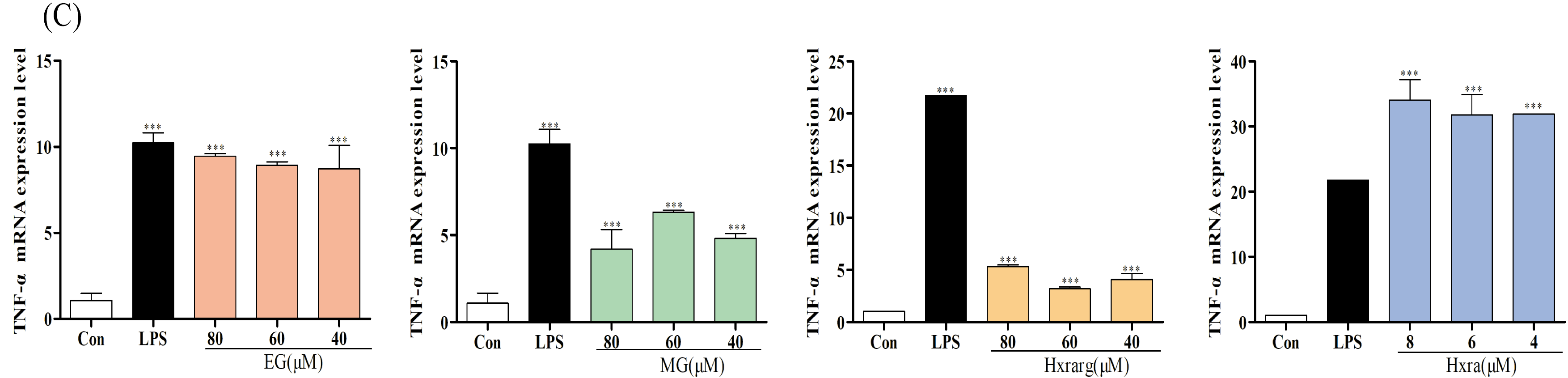

Supplement: Supplementary file 3 [file Data_Sheet_3.ZIP › Original Data/Fig. 6/TNF-a┴ mRNA/combined diagram.tif]

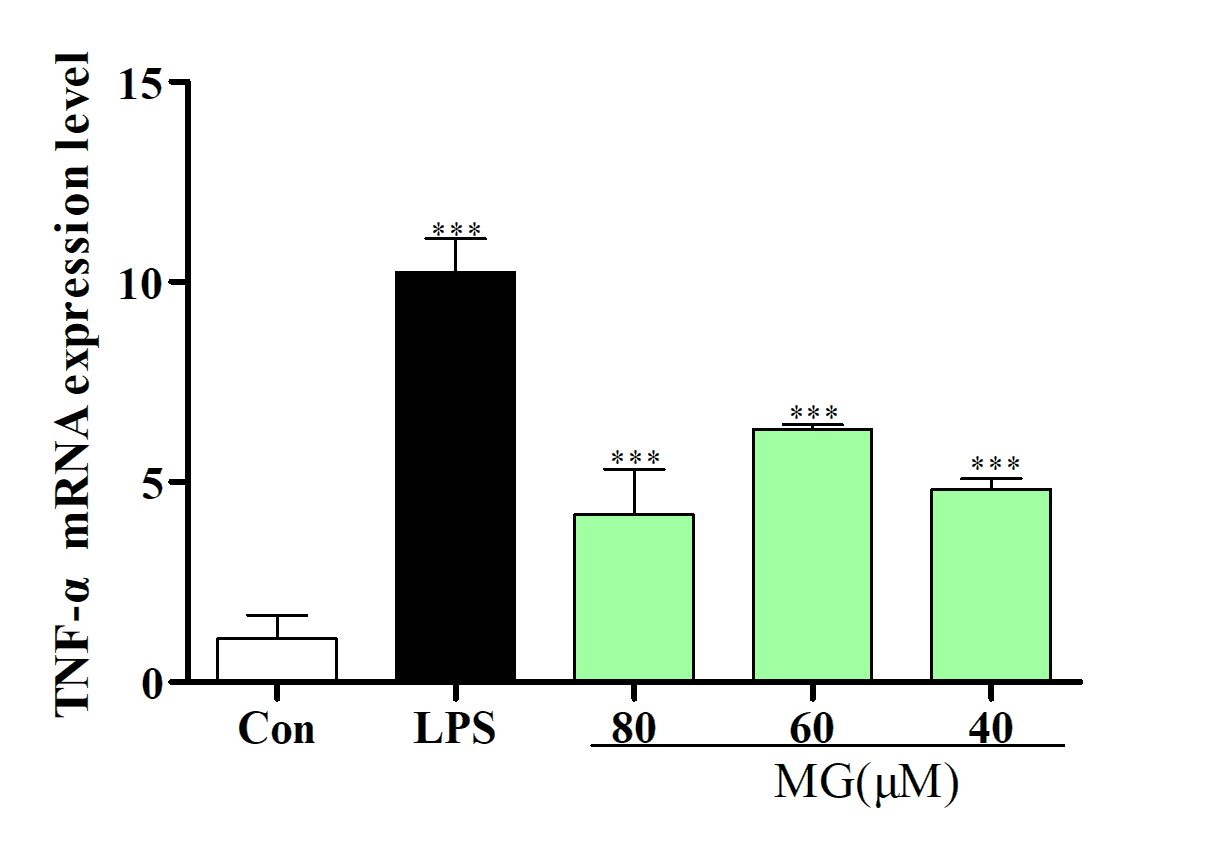

Supplement: Supplementary file 3 [file Data_Sheet_3.ZIP › Original Data/Fig. 6/TNF-a┴ mRNA/ú═ú╟-TNF.tif]

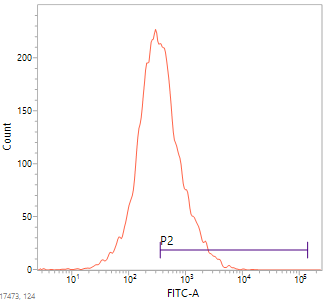

Supplement: Supplementary file 3 [file Data_Sheet_3.ZIP › Original Data/Fig. 7/EG/40-EG.png]

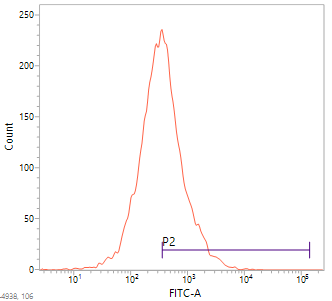

Supplement: Supplementary file 3 [file Data_Sheet_3.ZIP › Original Data/Fig. 7/EG/60-EG.png]

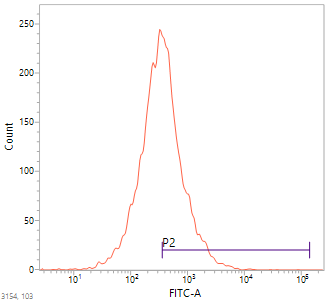

Supplement: Supplementary file 3 [file Data_Sheet_3.ZIP › Original Data/Fig. 7/EG/80-EG.png]

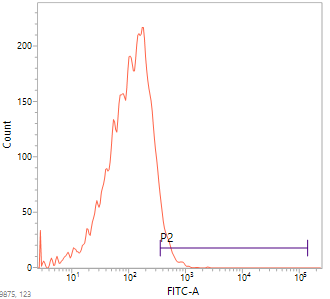

Supplement: Supplementary file 3 [file Data_Sheet_3.ZIP › Original Data/Fig. 7/EG/Control1.png]

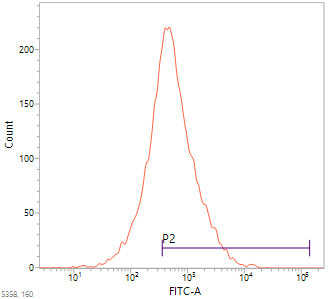

Supplement: Supplementary file 3 [file Data_Sheet_3.ZIP › Original Data/Fig. 7/EG/LPS1.png]

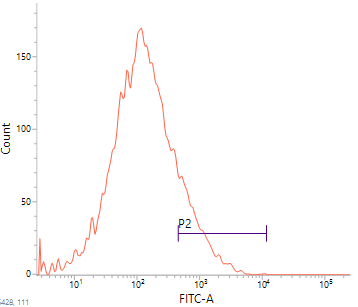

Supplement: Supplementary file 3 [file Data_Sheet_3.ZIP › Original Data/Fig. 7/HXRA/4-HXRA .png]

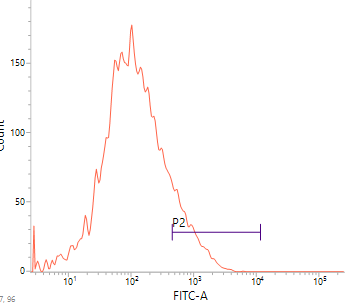

Supplement: Supplementary file 3 [file Data_Sheet_3.ZIP › Original Data/Fig. 7/HXRA/6-HXRA .png]

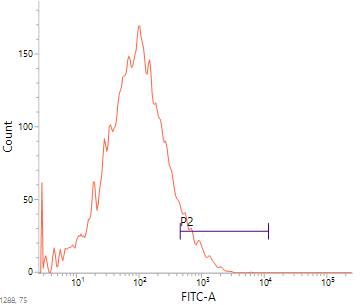

Supplement: Supplementary file 3 [file Data_Sheet_3.ZIP › Original Data/Fig. 7/HXRA/8-HXRA .png]

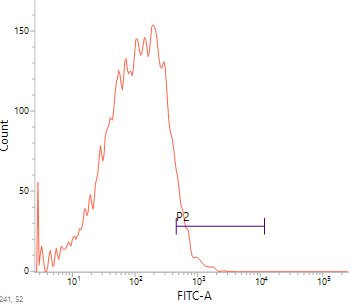

Supplement: Supplementary file 3 [file Data_Sheet_3.ZIP › Original Data/Fig. 7/HXRA/Control4.png]

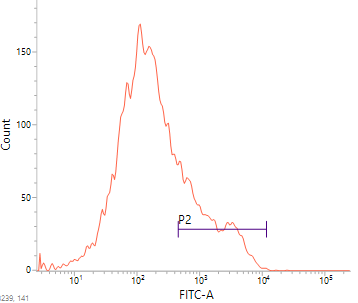

Supplement: Supplementary file 3 [file Data_Sheet_3.ZIP › Original Data/Fig. 7/HXRA/LPS4.png]

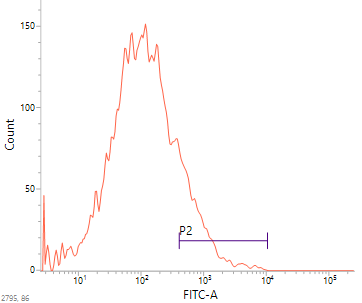

Supplement: Supplementary file 3 [file Data_Sheet_3.ZIP › Original Data/Fig. 7/HXRARG/40-HXRARG.png]

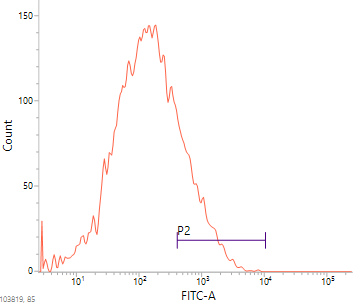

Supplement: Supplementary file 3 [file Data_Sheet_3.ZIP › Original Data/Fig. 7/HXRARG/60-HXRARG.png]

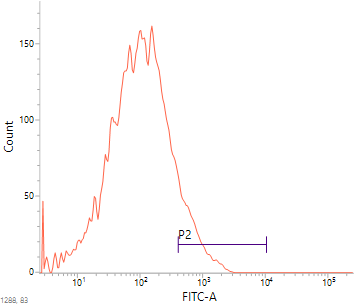

Supplement: Supplementary file 3 [file Data_Sheet_3.ZIP › Original Data/Fig. 7/HXRARG/80-HXRARG.png]

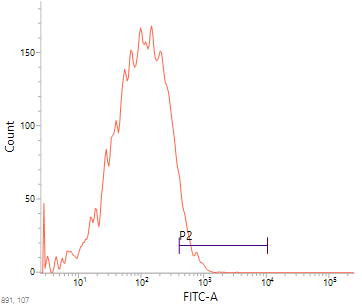

Supplement: Supplementary file 3 [file Data_Sheet_3.ZIP › Original Data/Fig. 7/HXRARG/Control3.png]

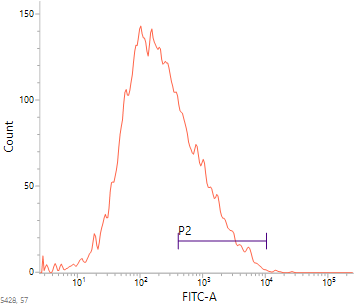

Supplement: Supplementary file 3 [file Data_Sheet_3.ZIP › Original Data/Fig. 7/HXRARG/LPS3.png]

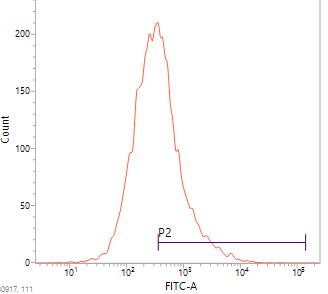

Supplement: Supplementary file 3 [file Data_Sheet_3.ZIP › Original Data/Fig. 7/MG/40-MG.png]

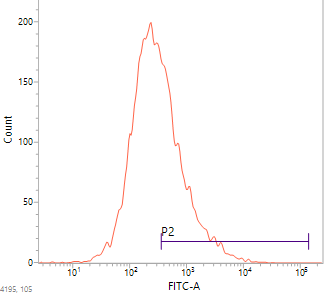

Supplement: Supplementary file 3 [file Data_Sheet_3.ZIP › Original Data/Fig. 7/MG/60-MG.png]

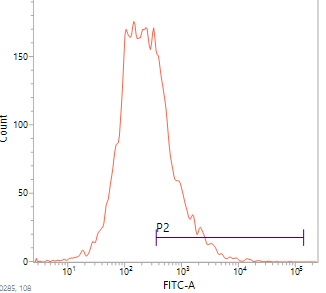

Supplement: Supplementary file 3 [file Data_Sheet_3.ZIP › Original Data/Fig. 7/MG/80-MG.png]

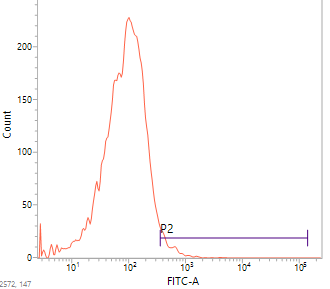

Supplement: Supplementary file 3 [file Data_Sheet_3.ZIP › Original Data/Fig. 7/MG/Control2.png]

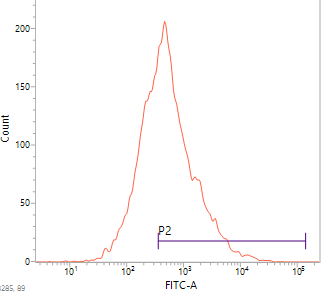

Supplement: Supplementary file 3 [file Data_Sheet_3.ZIP › Original Data/Fig. 7/MG/LPS2.png]

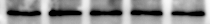

Supplement: Supplementary file 3 [file Data_Sheet_3.ZIP › Original Data/Fig. 8/EG/IKBa.tif]

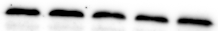

Supplement: Supplementary file 3 [file Data_Sheet_3.ZIP › Original Data/Fig. 8/EG/P65.tif]

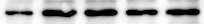

Supplement: Supplementary file 3 [file Data_Sheet_3.ZIP › Original Data/Fig. 8/EG/Pp65.tif]

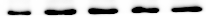

Supplement: Supplementary file 3 [file Data_Sheet_3.ZIP › Original Data/Fig. 8/EG/Ppikba.tif]

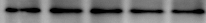

Supplement: Supplementary file 3 [file Data_Sheet_3.ZIP › Original Data/Fig. 8/EG/a┬-actin.tif]

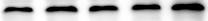

Supplement: Supplementary file 3 [file Data_Sheet_3.ZIP › Original Data/Fig. 8/HXRA/Ikba┴.tif]

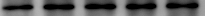

Supplement: Supplementary file 3 [file Data_Sheet_3.ZIP › Original Data/Fig. 8/HXRA/P65.tif]

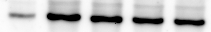

Supplement: Supplementary file 3 [file Data_Sheet_3.ZIP › Original Data/Fig. 8/HXRA/p-Ikba┴.tif]

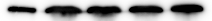

Supplement: Supplementary file 3 [file Data_Sheet_3.ZIP › Original Data/Fig. 8/HXRA/p-P65.tif]

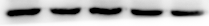

Supplement: Supplementary file 3 [file Data_Sheet_3.ZIP › Original Data/Fig. 8/HXRA/a┬-actin.tif]

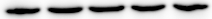

Supplement: Supplementary file 3 [file Data_Sheet_3.ZIP › Original Data/Fig. 8/HXRARG/IKba┴.tif]

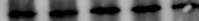

Supplement: Supplementary file 3 [file Data_Sheet_3.ZIP › Original Data/Fig. 8/HXRARG/P65.tif]

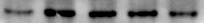

Supplement: Supplementary file 3 [file Data_Sheet_3.ZIP › Original Data/Fig. 8/HXRARG/p-IKba┴.tif]

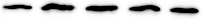

Supplement: Supplementary file 3 [file Data_Sheet_3.ZIP › Original Data/Fig. 8/HXRARG/p-P65.tif]

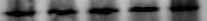

Supplement: Supplementary file 3 [file Data_Sheet_3.ZIP › Original Data/Fig. 8/HXRARG/a┬-actin.tif]

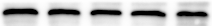

Supplement: Supplementary file 3 [file Data_Sheet_3.ZIP › Original Data/Fig. 8/MG/Ikba.tif]

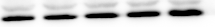

Supplement: Supplementary file 3 [file Data_Sheet_3.ZIP › Original Data/Fig. 8/MG/P65.tif]

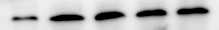

Supplement: Supplementary file 3 [file Data_Sheet_3.ZIP › Original Data/Fig. 8/MG/Pikba.tif]

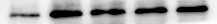

Supplement: Supplementary file 3 [file Data_Sheet_3.ZIP › Original Data/Fig. 8/MG/Pp65.tif]

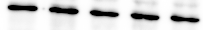

Supplement: Supplementary file 3 [file Data_Sheet_3.ZIP › Original Data/Fig. 8/MG/a┬-actin.tif]

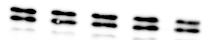

Supplement: Supplementary file 3 [file Data_Sheet_3.ZIP › Original Data/Fig. 9/EG/Erk.tif]

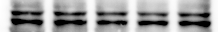

Supplement: Supplementary file 3 [file Data_Sheet_3.ZIP › Original Data/Fig. 9/EG/JNK.tif]

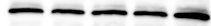

Supplement: Supplementary file 3 [file Data_Sheet_3.ZIP › Original Data/Fig. 9/EG/P38.tif]

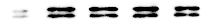

Supplement: Supplementary file 3 [file Data_Sheet_3.ZIP › Original Data/Fig. 9/EG/p-Erk.tif]

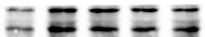

Supplement: Supplementary file 3 [file Data_Sheet_3.ZIP › Original Data/Fig. 9/EG/p-JNK.tif]

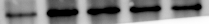

Supplement: Supplementary file 3 [file Data_Sheet_3.ZIP › Original Data/Fig. 9/EG/p-P38.tif]

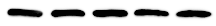

Supplement: Supplementary file 3 [file Data_Sheet_3.ZIP › Original Data/Fig. 9/EG/a┬-actin.tif]

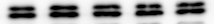

Supplement: Supplementary file 3 [file Data_Sheet_3.ZIP › Original Data/Fig. 9/HXRA/Erk.tif]

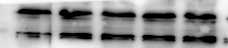

Supplement: Supplementary file 3 [file Data_Sheet_3.ZIP › Original Data/Fig. 9/HXRA/JNK.tif]

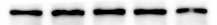

Supplement: Supplementary file 3 [file Data_Sheet_3.ZIP › Original Data/Fig. 9/HXRA/P38.tif]

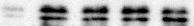

Supplement: Supplementary file 3 [file Data_Sheet_3.ZIP › Original Data/Fig. 9/HXRA/p-Erk.tif]

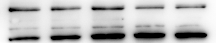

Supplement: Supplementary file 3 [file Data_Sheet_3.ZIP › Original Data/Fig. 9/HXRA/p-JNK.tif]

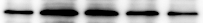

Supplement: Supplementary file 3 [file Data_Sheet_3.ZIP › Original Data/Fig. 9/HXRA/p-P38.tif]

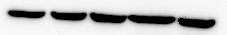

Supplement: Supplementary file 3 [file Data_Sheet_3.ZIP › Original Data/Fig. 9/HXRA/a┬-actin.tif]

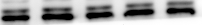

Supplement: Supplementary file 3 [file Data_Sheet_3.ZIP › Original Data/Fig. 9/HXRARG/Erk.tif]

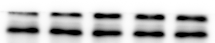

Supplement: Supplementary file 3 [file Data_Sheet_3.ZIP › Original Data/Fig. 9/HXRARG/JNK.tif]

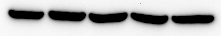

Supplement: Supplementary file 3 [file Data_Sheet_3.ZIP › Original Data/Fig. 9/HXRARG/MAPK-Actin.tif]

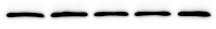

Supplement: Supplementary file 3 [file Data_Sheet_3.ZIP › Original Data/Fig. 9/HXRARG/P38.tif]

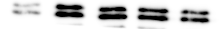

Supplement: Supplementary file 3 [file Data_Sheet_3.ZIP › Original Data/Fig. 9/HXRARG/p-Erk.tif]

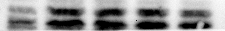

Supplement: Supplementary file 3 [file Data_Sheet_3.ZIP › Original Data/Fig. 9/HXRARG/p-JNK.tif]

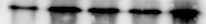

Supplement: Supplementary file 3 [file Data_Sheet_3.ZIP › Original Data/Fig. 9/HXRARG/p-P38.tif]

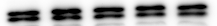

Supplement: Supplementary file 3 [file Data_Sheet_3.ZIP › Original Data/Fig. 9/MG/Erk.tif]

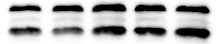

Supplement: Supplementary file 3 [file Data_Sheet_3.ZIP › Original Data/Fig. 9/MG/JNK.tif]

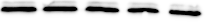

Supplement: Supplementary file 3 [file Data_Sheet_3.ZIP › Original Data/Fig. 9/MG/P38.tif]

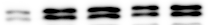

Supplement: Supplementary file 3 [file Data_Sheet_3.ZIP › Original Data/Fig. 9/MG/p-Erk.tif]

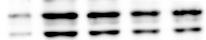

Supplement: Supplementary file 3 [file Data_Sheet_3.ZIP › Original Data/Fig. 9/MG/p-JNK.tif]

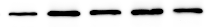

Supplement: Supplementary file 3 [file Data_Sheet_3.ZIP › Original Data/Fig. 9/MG/p-P38.tif]

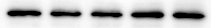

Supplement: Supplementary file 3 [file Data_Sheet_3.ZIP › Original Data/Fig. 9/MG/a┬-antin.tif]
