# Supplementary material for: The Immunomodulatory Effects of Active Ingredients From Nigella sativa in RAW264.7 Cells Through NF-κB/MAPK Signaling Pathways
Source: Front Nutr. 2022 May 31;9:899797. doi: 10.3389/fnut.2022.899797 (PMC9194833; doi:10.3389/fnut.2022.899797)
Supplement: Supplementary file 3 [file Data_Sheet_3.ZIP › Original Data/Fig. 7/EG/New Rich Text Document.rtf]

Statistics
Name	Events	% Parent	% Grandparent	% Total	FSC-A Mean	SSC-A Mean	
Control:All Events	100,552	***	***	100.00	57,901	46,760	
Control:P1	10,000	9.95	***	9.95	114,214	94,009	
Control:P2	495	4.95	0.49	0.49	125,618	119,574	
LPS:All Events	43,537	***	***	100.00	71,799	79,822	
LPS:P1	10,000	22.97	***	22.97	101,253	108,021	
LPS:P2	6,583	65.83	15.12	15.12	102,892	113,638	
F1:All Events	39,355	***	***	100.00	75,673	82,583	
F1:P1	10,000	25.41	***	25.41	103,687	109,849	
F1:P2	4,776	47.76	12.14	12.14	107,289	118,939	
F2:All Events	39,911	***	***	100.00	74,788	81,020	
F2:P1	10,000	25.06	***	25.06	102,979	108,664	
F2:P2	4,745	47.45	11.89	11.89	106,692	118,386	
F3:All Events	37,532	***	***	100.00	78,520	86,649	
F3:P1	10,000	26.64	***	26.64	107,151	115,897	
F3:P2	4,652	46.52	12.39	12.39	110,415	124,018	
