# Supplementary material for: The Immunomodulatory Effects of Active Ingredients From Nigella sativa in RAW264.7 Cells Through NF-κB/MAPK Signaling Pathways
Source: Front Nutr. 2022 May 31;9:899797. doi: 10.3389/fnut.2022.899797 (PMC9194833; doi:10.3389/fnut.2022.899797)
Supplement: Supplementary file 3 [file Data_Sheet_3.ZIP › Original Data/Fig. 7/HXRA/New Rich Text Document.rtf]

Statistics
Name	Events	% Parent	% Grandparent	% Total	FSC-A Mean	SSC-A Mean	
Control:All Events	84,952	***	***	100.00	36,547	20,425	
Control:P1	10,000	11.77	***	11.77	67,200	42,220	
Control:P2	598	5.98	0.70	0.70	86,625	62,804	
LPS+20:All Events	56,091	***	***	100.00	40,029	38,012	
LPS+20:P1	10,000	17.83	***	17.83	59,618	58,466	
LPS+20:P2	714	7.14	1.27	1.27	62,793	59,744	
LPS+10:All Events	51,163	***	***	100.00	40,550	39,661	
LPS+10:P1	10,000	19.55	***	19.55	62,357	63,163	
LPS+10:P2	1,202	12.02	2.35	2.35	64,400	65,256	
LPS+5:All Events	50,180	***	***	100.00	41,216	40,174	
LPS+5:P1	10,000	19.93	***	19.93	61,071	61,545	
LPS+5:P2	1,448	14.48	2.89	2.89	63,299	63,218	
LPS:All Events	48,941	***	***	100.00	40,968	44,810	
LPS:P1	10,000	20.43	***	20.43	59,798	63,305	
LPS:P2	2,421	24.21	4.95	4.95	61,759	60,915	
