# Supplementary material for: The Immunomodulatory Effects of Active Ingredients From Nigella sativa in RAW264.7 Cells Through NF-κB/MAPK Signaling Pathways
Source: Front Nutr. 2022 May 31;9:899797. doi: 10.3389/fnut.2022.899797 (PMC9194833; doi:10.3389/fnut.2022.899797)
Supplement: Supplementary file 3 [file Data_Sheet_3.ZIP › Original Data/Fig. 7/HXRARG/New Rich Text Document.rtf]

Statistics
Name	Events	% Parent	% Grandparent	% Total	FSC-A Mean	SSC-A Mean	
Control:All Events	44,541	***	***	100.00	42,169	25,079	
Control:P1	10,000	22.45	***	22.45	72,992	48,118	
Control:P2	552	5.52	1.24	1.24	97,189	79,976	
LPS:All Events	45,298	***	***	100.00	41,147	38,008	
LPS:P1	10,000	22.08	***	22.08	57,114	52,619	
LPS:P2	2,958	29.58	6.53	6.53	58,666	51,130	
LPS+20:All Events	63,328	***	***	100.00	38,804	28,800	
LPS+20:P1	10,000	15.79	***	15.79	60,249	46,215	
LPS+20:P2	992	9.92	1.57	1.57	64,166	50,380	
LPS+10:All Events	52,473	***	***	100.00	40,536	34,424	
LPS+10:P1	10,000	19.06	***	19.06	61,991	55,400	
LPS+10:P2	1,941	19.41	3.70	3.70	63,939	56,438	
LPS+5:All Events	43,202	***	***	100.00	43,126	35,184	
LPS+5:P1	10,000	23.15	***	23.15	61,795	53,293	
LPS+5:P2	1,437	14.37	3.33	3.33	64,290	57,443	
