# Supplementary material for: The Immunomodulatory Effects of Active Ingredients From Nigella sativa in RAW264.7 Cells Through NF-κB/MAPK Signaling Pathways
Source: Front Nutr. 2022 May 31;9:899797. doi: 10.3389/fnut.2022.899797 (PMC9194833; doi:10.3389/fnut.2022.899797)
Supplement: Supplementary file 3 [file Data_Sheet_3.ZIP › Original Data/Fig. 7/MG/New Rich Text Document.rtf]

Statistics
Name	Events	% Parent	% Grandparent	% Total	FSC-A Mean	SSC-A Mean	
Control:All Events	53,892	***	***	100.00	63,605	54,033	
Control:P1	10,000	18.56	***	18.56	117,427	101,908	
Control:P2	496	4.96	0.92	0.92	132,929	129,921	
LPS:All Events	51,301	***	***	100.00	73,595	82,440	
LPS:P1	10,000	19.49	***	19.49	108,942	117,654	
LPS:P2	7,018	70.18	13.68	13.68	110,501	121,823	
N1:All Events	58,401	***	***	100.00	73,598	80,749	
N1:P1	10,000	17.12	***	17.12	113,301	123,307	
N1:P2	4,004	40.04	6.86	6.86	113,759	130,085	
N2:All Events	45,015	***	***	100.00	79,591	88,739	
N2:P1	10,000	22.21	***	22.21	115,665	126,517	
N2:P2	4,833	48.33	10.74	10.74	118,328	133,919	
N3:All Events	45,968	***	***	100.00	78,307	86,282	
N3:P1	10,000	21.75	***	21.75	115,355	125,094	
N3:P2	5,570	55.70	12.12	12.12	118,366	131,638	
